# Porphyromonas gingivalis Outer Membrane Vesicles Promote Apoptosis via msRNA-Regulated DNA Methylation in Periodontitis

**DOI:** 10.1128/spectrum.03288-22

**Published:** 2023-01-11

**Authors:** Ruyi Fan, Yi Zhou, Xu Chen, Xianmei Zhong, Fanzhen He, Wenzao Peng, Lu Li, Xiaoqian Wang, Yan Xu

**Affiliations:** a Department of Periodontics, the Affiliated Stomatological Hospital of Nanjing Medical University, Nanjing, China; b Jiangsu Province Key Laboratory of Oral Diseases, Nanjing Medical University, Nanjing, China; c Jiangsu Province Engineering Research Center of Stomatological Translational Medicine, Nanjing, China; d Department of Periodontics, Taizhou Stomatological Hospital, Taizhou, China; Griffith University; University of Florida

**Keywords:** outer membrane vesicles, *Porphyromonas gingivalis*, periodontitis, apoptosis, DNA methylation

## Abstract

The outer membrane vesicles (OMVs) produced by Porphyromonas gingivalis contain a variety of bioactive molecules that may be involved in the progression of periodontitis. However, the participation of P. gingivalis OMVs in the development of periodontitis has not been elucidated. Here, we isolated P. gingivalis OMVs and confirmed their participation in periodontitis both *in vivo* and *in vitro*. Microcomputed tomography (micro-CT) and histological analysis showed that under stimulation with P. gingivalis OMVs, the alveolar bone of rats was significantly resorbed in vivo. We found that P. gingivalis OMVs were taken up by human periodontal ligament cells ([hPDLCs]) *in vitro*, which subsequently resulted in apoptosis and inflammatory cytokine release, which was accomplished by the microRNA-size small RNA (msRNA) sRNA45033 in the P. gingivalis OMVs. Through bioinformatics analysis and screening of target genes, chromobox 5 (CBX5) was identified as the downstream target of screened-out sRNA45033. Using a dual-luciferase reporter assay, overexpression, and knockdown methods, sRNA45033 was confirmed to target CBX5 to regulate hPDLC apoptosis. In addition, CUT&Tag (cleavage under targets and tagmentation) analysis confirmed the mechanism that CBX5 regulates apoptosis through the methylation of p53 DNA. Collectively, these findings indicate that the role of P. gingivalis OMVs is immunologically relevant and related to bacterial virulence during the development of periodontitis.

**IMPORTANCE** P. gingivalis is a bacterium often associated with periodontitis. This study demonstrates that (i) sRNA45033 in P. gingivalis OMVs targets CBX5, (ii) CBX5 regulates the methylation of p53 DNA and its expression, which is associated with apoptosis, and (iii) a novel mechanism of interaction between hosts and pathogens is mediated by OMVs in the occurrence of periodontitis.

## INTRODUCTION

As the sixth most common human disease, chronic periodontitis is a major public health issue, with a high prevalence of 45% to 50% (1). The prevalent oral disease is usually characterized by chronic inflammation, which results from host inflammatory responses against Gram-negative anaerobic bacteria (2). An imbalance in host-bacterium interaction results in the disruption of periodontal homeostasis, which leads to the initiation and progression of periodontitis (3). The dysbiotic microbial environment triggered by the pathogen releases proinflammatory factors into the periodontium, which causes chronic inflammation and ultimately results in the destruction of soft tissue support, gingival recession, bone resorption, and the loss of teeth (4, 5).

The Gram-negative anaerobic bacterium Porphyromonas gingivalis is considered the keystone pathogen in the development of periodontitis (6). P. gingivalis can colonize into the subgingival pocket to initiate the periodontitis (7), producing virulence factors, like gingipains and fimbrillin, to further damage the periodontal tissue (8), disrupt the balance of resident microbiota, and impair the host immune system (9).

Outer membrane vesicles (OMVs) are composed of a single lipid bilayer (which is derived from the bacterial outer membrane), which originates from all Gram-negative bacteria (10). P. gingivalis is also the most prolific OMV producer, and the sizes of the vesicles range from 50 to 400 nm (11). P. gingivalis OMVs contain virulence factors, like fimbriae, gingipains, and lipopolysaccharide (LPS) (12). P. gingivalis OMVs are highly inflammatory, with the ability to regulate neutrophils and macrophages and invade oral epithelial cells (13–15). These findings implicate P. gingivalis OMVs in important roles in periodontitis.

OMVs contains proteins, lipid, nucleic acids, and other biofunctional molecules, among which, small RNAs (sRNAs) in the OMVs have received attention for their potential gene regulatory functions as interspecies communication molecules (16, 17). There has been evidence that various bacteria utilize sRNAs interacting with RNA-induced silencing complex (RISC) to inhibit host immunity genes and promote intracellular survival (18–20). sRNAs enclosed and protected by OMVs could be transported into host cells and then regulate gene expression or immune responses (16, 21–23). Several bacteria, including periodontal pathogens (i.e., P. gingivalis, Aggregatibacter actinomycetemcomitans, and Treponema denticola) also produce a novel class of sRNAs of microRNA (miRNA) size (msRNAs) (17, 24, 25), which could be taken up by host cells, such as Jurkat T cells. Choi et al. demonstrated that after transfection of synthetic sRNA, immune responses were altered by decreasing levels of interleukin-5 (IL-5), IL-13, and IL-15 (17). In another study, the authors found sRNAs in *A. actinomycetemitans* OMVs increased tumor necrosis factor alpha (TNF-α) via the Toll-like receptor 8 (TLR-8) and NF-κB signaling pathways (26). sRNA23392 in the P. gingivalis OMVs was found targeting desmocollin-2 to promote oral squamous cell carcinoma migration and invasion (11). Bioinformatic analysis found that reads from P. gingivalis, A. actinomycetemcomitans, and T. denticola overlap some human genome regulatory regions and are aligned against some histone mark regions, which suggest possible function similar to long noncoding RNAs and epigenetic roles (27). However, extensive studies of OMVs and their sRNAs need to be performed to understand their roles, which could provide more insight into the development of diseases like periodontitis.

The eukaryotic genome constitutes transcriptionally active euchromatin and transcriptionally silent heterochromatin. Chromobox 5 (CBX5), also known as heterochromatin protein 1 alpha, is an architectural protein that binds DNA to form and maintain heterochromatin through association with the protein H3K9me3 (trimethylated of lysine 9 of histone H3) (28–30). Recent studies have found that CBX5 was related to heterochromatin-repressed inflammatory response, apoptosis, and death receptor signaling (31). CBX5 methylates H3K9 lysines, which form heterochromatin to silence relevant genes such as *IFNL1*, *CXCL10*, *CXCL11*, and *USP18* (32). Apoptosis is a programmed cell death process that responds to diverse stress situations. p53 is a crucial protein that induces apoptosis in response to various stimuli (33).

The participation of P. gingivalis OMVs in the development of periodontitis has not been elucidated. Here, we report that P. gingivalis OMVs were taken up by human periodontal ligament cells (hPDLCs), subsequently resulting in apoptosis and inflammatory cytokine release. This process was accomplished by the sRNA45033 in the P. gingivalis OMVs targeting CBX5. We also report that P. gingivalis OMVs induced apoptosis through p53 expression regulated by CBX5-associated H3K9me3. Therefore, our findings suggest that a novel mechanism of interaction between host, pathogens, and OMVs plays an important role in the occurrence of periodontitis.

## RESULTS

### Morphological characterization of P. gingivalis OMVs.

**FIG 1:**
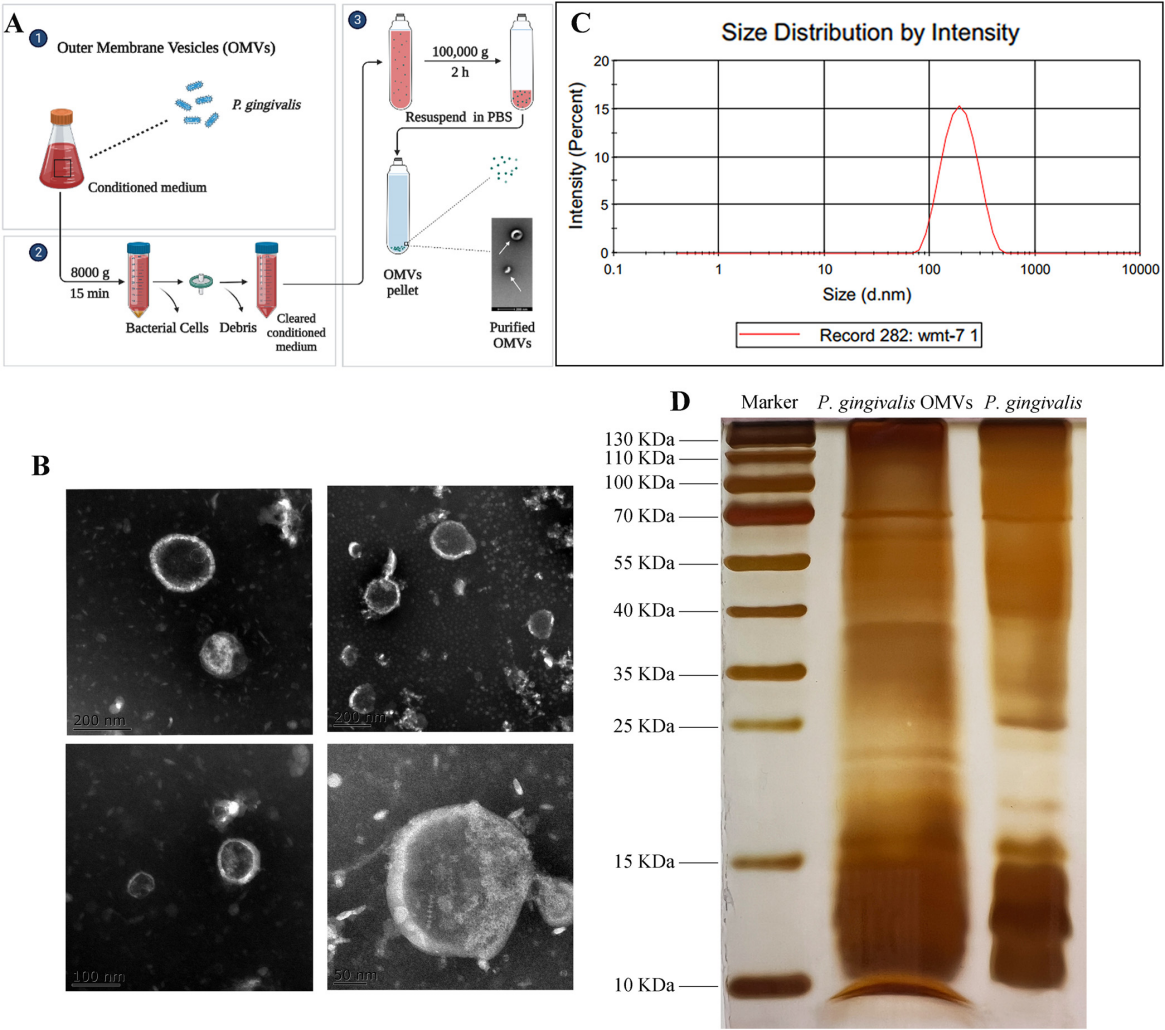
Morphological characterization of P. gingivalis OMVs. (A) Illustration of OMV preparation. (B) The images of isolated P. gingivalis OMVs from culture supernatant were captured by TEM. Scale bars = 200 nm, 100 nm, and 50 nm. (C) The particle size distribution of isolated P. gingivalis OMVs was measured by dynamic light scattering. (D) The protein contents of P. gingivalis OMVs and P. gingivalis are shown by silver staining.

We collected OMVs from P. gingivalis culture supernatant, characterized the isolated P. gingivalis OMVs using transmission electron microscopy (TEM), and measured their particle size distribution. The TEM images revealed that P. gingivalis OMVs are considerably round and oval (Fig. 1B), with a broad size range (Fig. 1C). The average diameter of the OMVs isolated from P. gingivalis cultures was 202.8 nm (Fig. 1C). Several protein bands of *P. gingivalis* OMVs were common to proteins of P. gingivalis according to silver staining (Fig. 1D) and Coomassie blue staining (see Fig. S1 in the supplemental material).

### P. gingivalis OMVs promote alveolar bone resorption *in vivo*.

**FIG 2:**
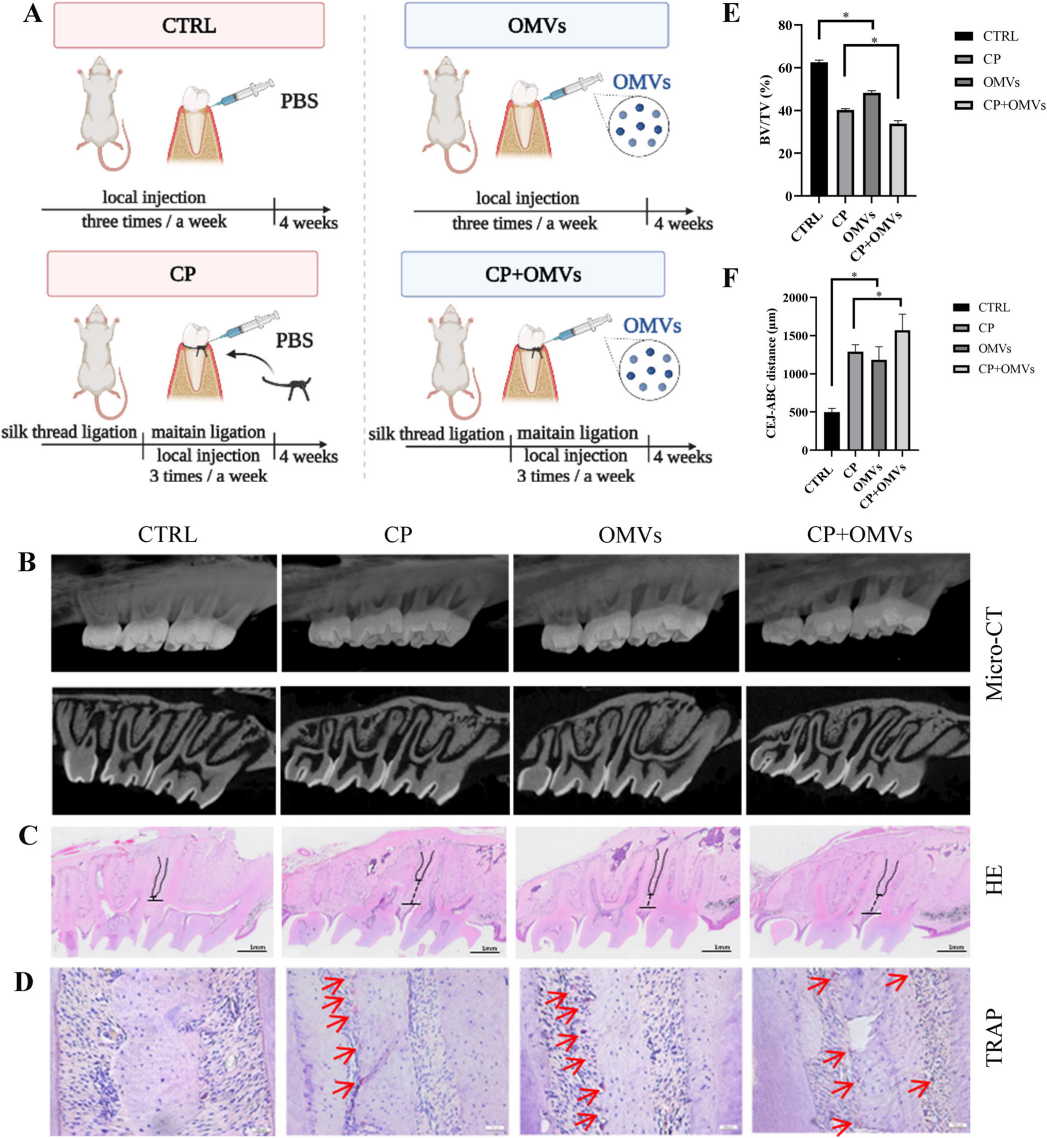
P. gingivalis OMVs promote alveolar bone resorption *in vivo*. (A) Schematic diagram of animal experiments. (B) The level of the rat alveolar bone resorption of four groups was measured by micro-CT and (C) H&E staining. The distances from the CEJ to ABC are marked in black. (D) TRAP staining. Red arrows indicate TRAP-positive surfaces. (E and F) The percentage of bone volume over tissue volume (BV/TV) and the distance from the cementoenamel junction (CEJ) to the alveolar bone crest (ABC) were calculated. Data are shown as mean ± SD with *n* = 5 or 6 rats per group. Data between two groups were compared using Student's *t* test. *, *P* < 0.05.

To understand the role of P. gingivalis OMVs, a periodontitis model of Sprague-Dawley (SD) rats was constructed. A three-dimensional (3D) reconstruction image of the rat alveolar bone showed significant bone resorption in the P. gingivalis OMV-treated rats (OMVs group) compared to the control rats (CTRL group), as well as the rats with chronic periodontitis treated with P. gingivalis OMVs (CP+OMVs group) compared to the untreated rats with chronic periodontitis (CP model group) (Fig. 2B). The 3D reconstruction images and volumetric measurements from microcomputed tomography (micro-CT) analysis revealed the distance from the cementoenamel junction (CEJ) to the alveolar bone crest (ABC) and the percentage of bone volume over tissue volume (BV/TV), which further confirmed the significant resorption of alveolar bone in the OMVs group (Fig. 2E and F). Morphometric measurements of H&E-stained tissue sections indicated similar results to micro-CT—the distances from the CEJ to ABC were greater in the OMVs group and CP+OMVs group, respectively (Fig. 2C). Tartrate-resistant acid phosphatase (TRAP)-positive surfaces in the OMVs and CP+OMVs groups were, respectively, increased compared to the control and CP model groups (Fig. 2D).

### P. gingivalis OMVs decreased cell viability in hPDLCs.

**FIG 3:**
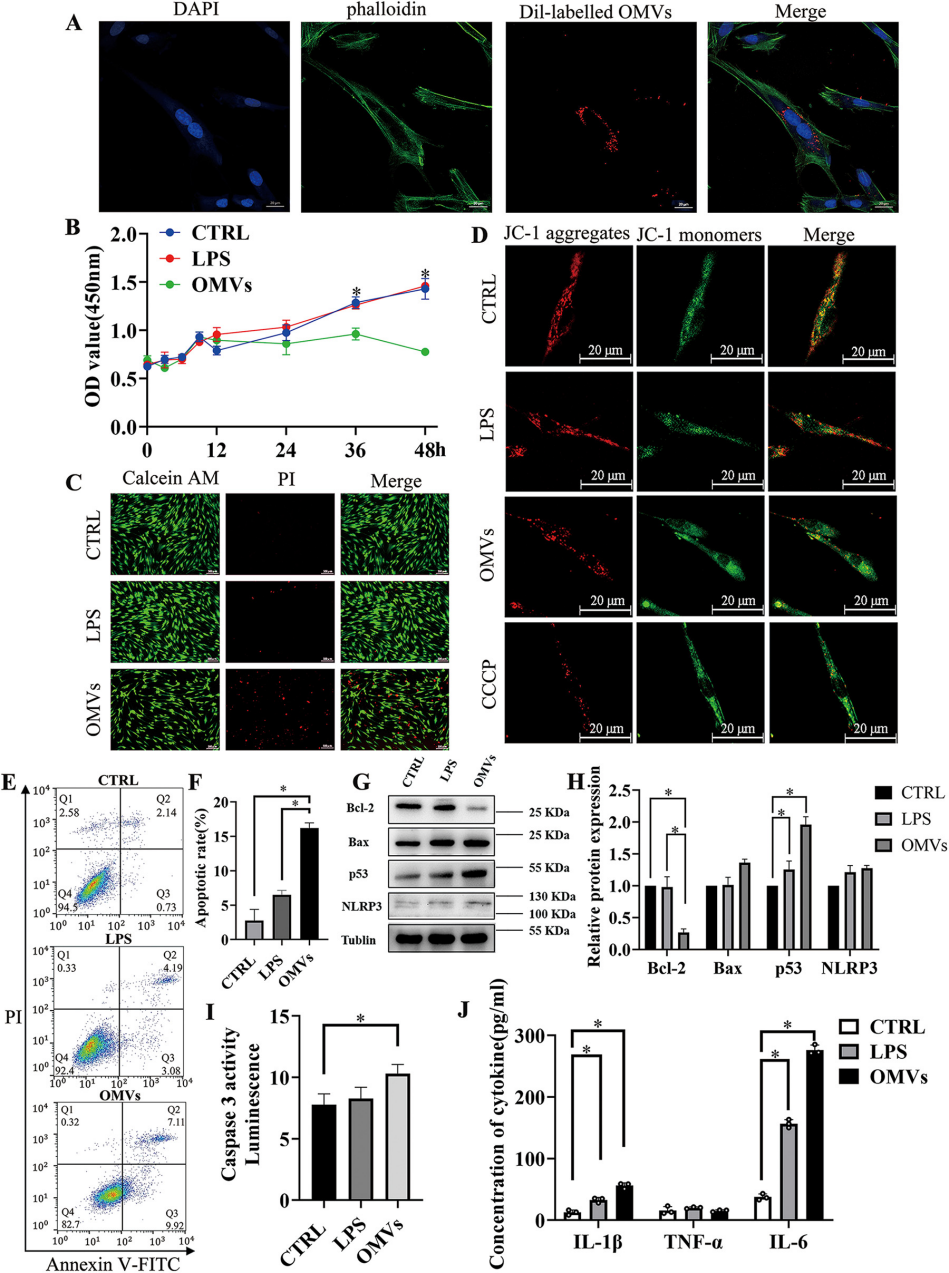
P. gingivalis OMVs decrease cell viability and promote apoptosis and inflammation in hPDLCs. (A) Endocytosis analysis demonstrated that the isolated P. gingivalis OMVs were taken up by the hPDLCs. (B) Cell proliferation of hPDLCs stimulated by the LPS or OMVs was measured with the CCK-8 assay. (C) PI staining image of control, LPS-treated, or OMV-treated hPDLCs. (D) The fluorescent probe JC-1 was used to measure mitochondrial membrane potential. CCCP, carbonyl cyanide *m*-chlorophenyl hydrazone. (E and F) Flow cytometry analysis of hPDLCs after being treated with LPS or OMVs. (G and H) Western blotting showed the expression of apoptosis-related proteins like BclII, Bax, p53, and inflammation-associated protein NLRP3 in hPDLCs treated with LPS or OMVs. (I) Caspase 3 activity was measured with the caspase-3 assay kit. (J) Secretion of different cytokines of hPDLCs measured by ELISA. Data are shown as mean ± SD. Data between two groups were compared using Student's *t* test. Cell experiments were conducted three times independently. *, *P* < 0.05.

The supernatant containing Dil (1,1′-dioctadecyl-3,3,3,3′-tetramethylindocarbocyanine perchlorate)-labeled P. gingivalis OMVs were cocultured with hPDLCs to demonstrate that P. gingivalis OMVs were incorporated into hPDLCs (Fig. 3A). Different from P. gingivalis lipopolysaccharide (LPS), P. gingivalis OMVs significantly decreased the viability of hPDLCs in a time- and concentration-dependent manner (Fig. 3B and Fig. S3). In the LIVE/DEAD viability/cytotoxicity assay, live cells were represented by green fluorescence, while dead cells were represented by red fluorescence in the fluorescence imaging experiments; therefore, propidium iodide (PI) staining also showed that P. gingivalis OMVs significantly increased cell death (Fig. 3C). Next, we examined whether P. gingivalis OMVs can induce mitochondrial dysfunction in hPDLCs. Generally, the decrease of mitochondrial membrane potential (MMP [ΔΨm]) is considered to be one of the indicators of mitochondrial dysfunction and an early feature of apoptotic cells. In this experiment, the changes in MMP were measured by using a fluorescent probe, JC-1. As shown in Fig. 3D, in the P. gingivalis OMV-stimulated cells, the red fluorescence decreased and the green fluorescence increased compared to control and LPS-stimulated cells, which suggested OMV-stimulated cells had lower mitochondrial membrane potential. These results suggested that P. gingivalis OMVs induced the apoptosis of hPDLCs.

### P. gingivalis OMVs promote apoptosis and inflammation in hPDLCs.

As flow cytometric analysis shows in Fig. 3E and F, the calculated cell death was considerably enhanced in cells treated with P. gingivalis OMVs versus the control and LPS groups. Furthermore, we evaluated the protein expression levels of three important mediators of apoptosis, including Bax, Bcl-2, p53, and caspase-3 activity. Western blotting showed that treatment with P. gingivalis OMVs resulted in a significant increase in the expression level of p53 and downregulation of antiapoptotic protein Bcl-2 compared to the control and LPS groups (Fig. 3G and H). Consistently, direct targets of p53 and proapoptotic proteins NOXA and PUMA (p53-upregulated modulator of apoptosis) were also increased after stimulation of P. gingivalis OMVs (Fig. S4A and B). Caspase-3 activity assay also showed that P. gingivalis OMVs resulted in a significant increase in the expression levels of caspase-3 versus the control (Fig. 3I). Levels of IL-1β, IL-6, and TNF-α were measured by enzyme-linked immunosorbent assay (ELISA) using a commercial kit. Relative to the negative control, the OMVs group had significantly increased levels of IL-1β and IL-6 (Fig. 3J). NLRP3 (NOD-, LRR-, and pyrin domain-containing protein 3) is a key component to form and activate the NLRP3 inflammasome, which results in the release of proinflammatory cytokines and promotes pyroptosis (34). Western blotting also showed that treatment of P. gingivalis OMVs resulted in a significant increase in the expression levels of NLRP3 (Fig. 3G and H). These results showed that the P. gingivalis OMVs promoted apoptosis and inflammation in hPDLCs.

### CBX5 is a functional target of sRNA45033 involved in hPDLC apoptosis.

**FIG 4:**
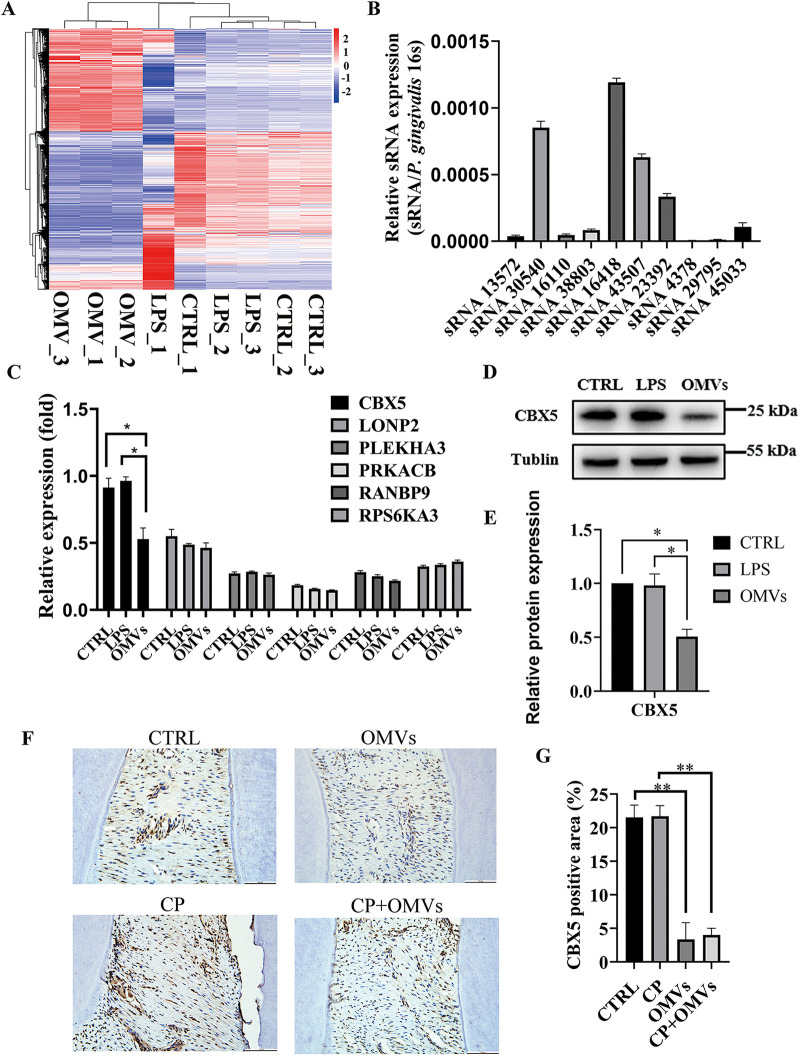
CBX5 is a functional target of sRNA45033 involved in hPDLC apoptosis. (A) Transcriptome sequencing analysis of hPDLCs administered P. gingivalis OMVs; (B) relative expression of msRNA in P. gingivalis OMVs; (C) possible target screening of sRNA45033; (D and E) CBX5 expression of hPDLCs treated with LPS or OMVs; (F and G) CBX5 immunochemistry analysis of the CTRL group, CP group, OMVs group, and CP+OMVs group. The positive areas in four independently chosen fields were counted and averaged. Data are shown as mean ± SD. Data between two groups were compared using Student's *t* test. Cell experiments were conducted three times independently. *, *P* < 0.05; **, *P* < 0.01.

The analysis of transcriptome sequencing results showed that P. gingivalis OMVs were involved in gene regulation, mRNA processing, endocytosis, ubiquitination, and the cell cycle process (Fig. 4A and Fig. S5). Data sets were uploaded to the Gene Expression Omnibus. Combined with previous research (17), Fig. 4B showed small RNAs secreted via P. gingivalis OMVs, in which sRNA30540, sRNA16418, sRNA43507, sRNA23392, and sRNA45033 were highly expressed. We predicted the functional targets of these small RNAs with relatively high expression through bioinformatic analysis (https://mrmicrot.imsi.athenarc.gr/?r=mrmicrot/index), and we found six possible differentially expressed targeted genes of these msRNAs (Fig. 4C). Then, targeted genes with high scores and associated with apoptosis were further validated by quantitative reverse transcription-PCR (qRT-PCR), Western blotting, and immunocytochemistry, and we finally screened out gene CBX5 (a downstream target of sRNA45033) and found that it was expressed at low levels in P. gingivalis OMV-stimulated hPDLCs and tissues (Fig. 4D to G).

### sRNA45033 directly bound to the 3′ UTR of CBX5 and regulate hPDLCs apoptosis.

**FIG 5:**
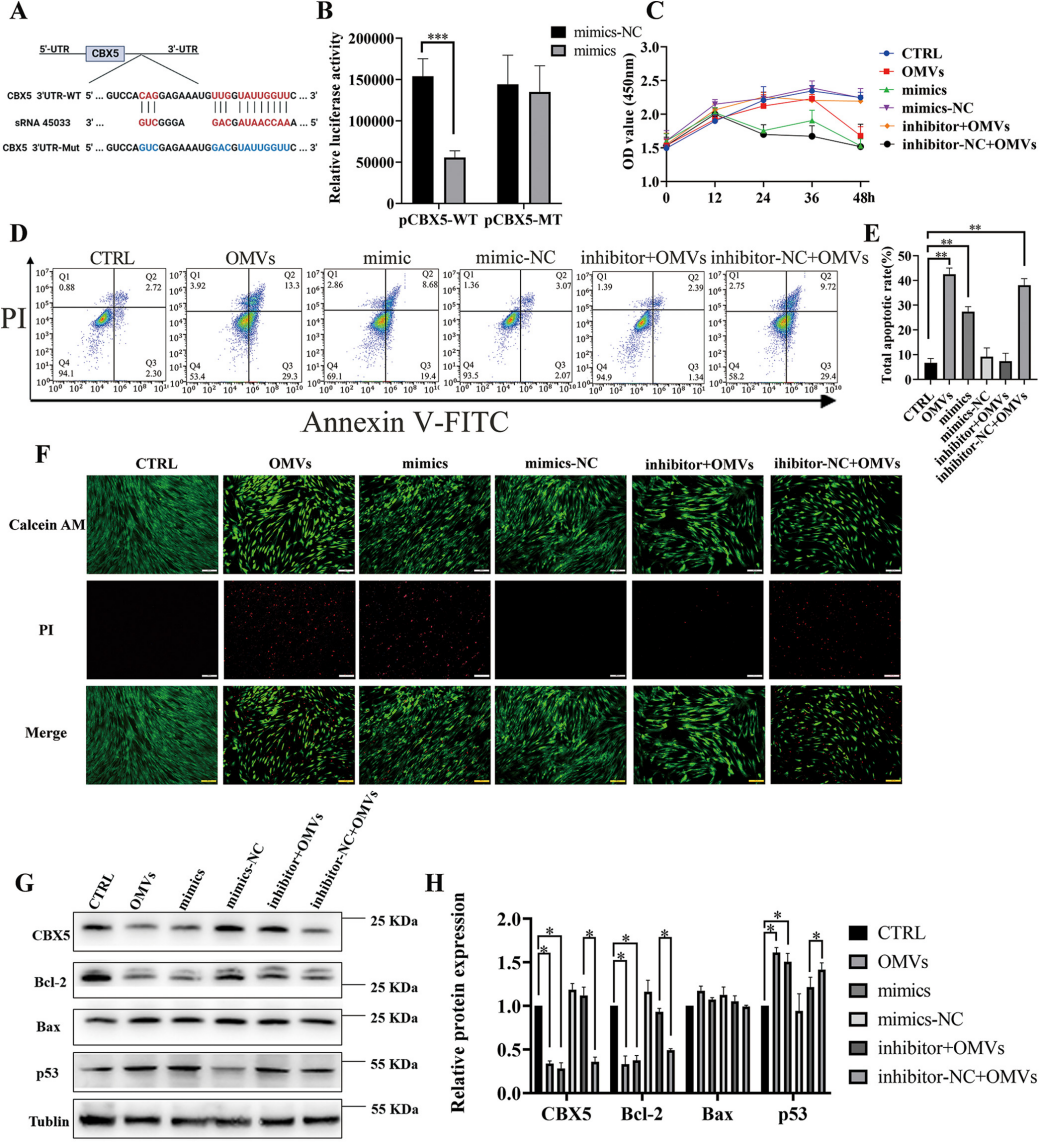
sRNA45033 directly bound to the 3′ UTR of CBX5 and regulates hPDLC apoptosis. (A) Schematic diagram of mutant strategy of the 3′ UTR of CBX5; (B) dual-luciferase analysis of plasmids with wild-type or mutant 3′ UTR of CBX5; (C) hPDLC proliferation in the OMVs, sRNA45033 mimics, mimics−NC, inhibitor+OMVs, and inhibitor−NC+OMVs groups measured by CCK-8 assay; (D and E) flow cytometry analysis; (F) PI staining; (G and H) Western blot and densitometric analyses of each group. Data are shown as mean ± SD. Data between two groups were compared using Student's *t* test. Cell experiments were conducted three times independently. *, *P* < 0.05; **, *P* < 0.01; ***, *P* < 0.001. NC indicates negative control.

To ascertain whether sRNA45033 directly binds to the 3′ untranslated region (UTR) of CBX5 and causes translational inhibition, CBX5 wild-type (pCBX5-WT) and mutant (pCBX5-Mut) plasmids were constructed. sRNA45033 mimics (but not the control group) significantly decreased the luciferase activity of the reporter containing the 3′ UTR wild type (WT) of CBX5 compared to the mutant counterpart (Fig. 5A and B). The luciferase assay confirmed that sRNA45033 targeted the 3′ UTR of CBX5 and reduced the expression of CBX5.

Next, we explored the role of sRNA45033 in cell apoptosis. The Cell Counting Kit-8 (CCK-8) assay results showed that sRNA45033 mimics in hPDLCs caused a significant decrease in the viability of hPDLCs, and inhibition of sRNA45033 dramatically reversed cell viability compared to the results observed in the P. gingivalis OMV-stimulated group (Fig. 5C). PI staining and flow cytometric analysis both showed that sRNA45033 mimics significantly increased cell apoptosis, while inhibition of sRNA45033 significantly reversed cell apoptosis compared to the results observed in the P. gingivalis OMV-stimulated group (Fig. 5D to F). sRNA45033 mimic-transfected cells displayed a similar pattern to the P. gingivalis OMV-stimulated group, the apoptosis marker p53 showed a significant increase in expression, while Bcl-2 showed a significant downregulation. The sRNA45033 inhibitor group displayed a reverse trend (Fig. 5G and H). PUMA and NOXA were also increased when treated with the sRNA45033 mimic and P. gingivalis OMVs and decreased when treated with sRNA45033 inhibitor (Fig. S4C and D). These results supported the hypothesis that sRNA45033 induces cell apoptosis by blocking CBX5 activity.

### CBX5 regulates apoptosis through p53 in P. gingivalis OMV-stimulated hPDLCs.

**FIG 6:**
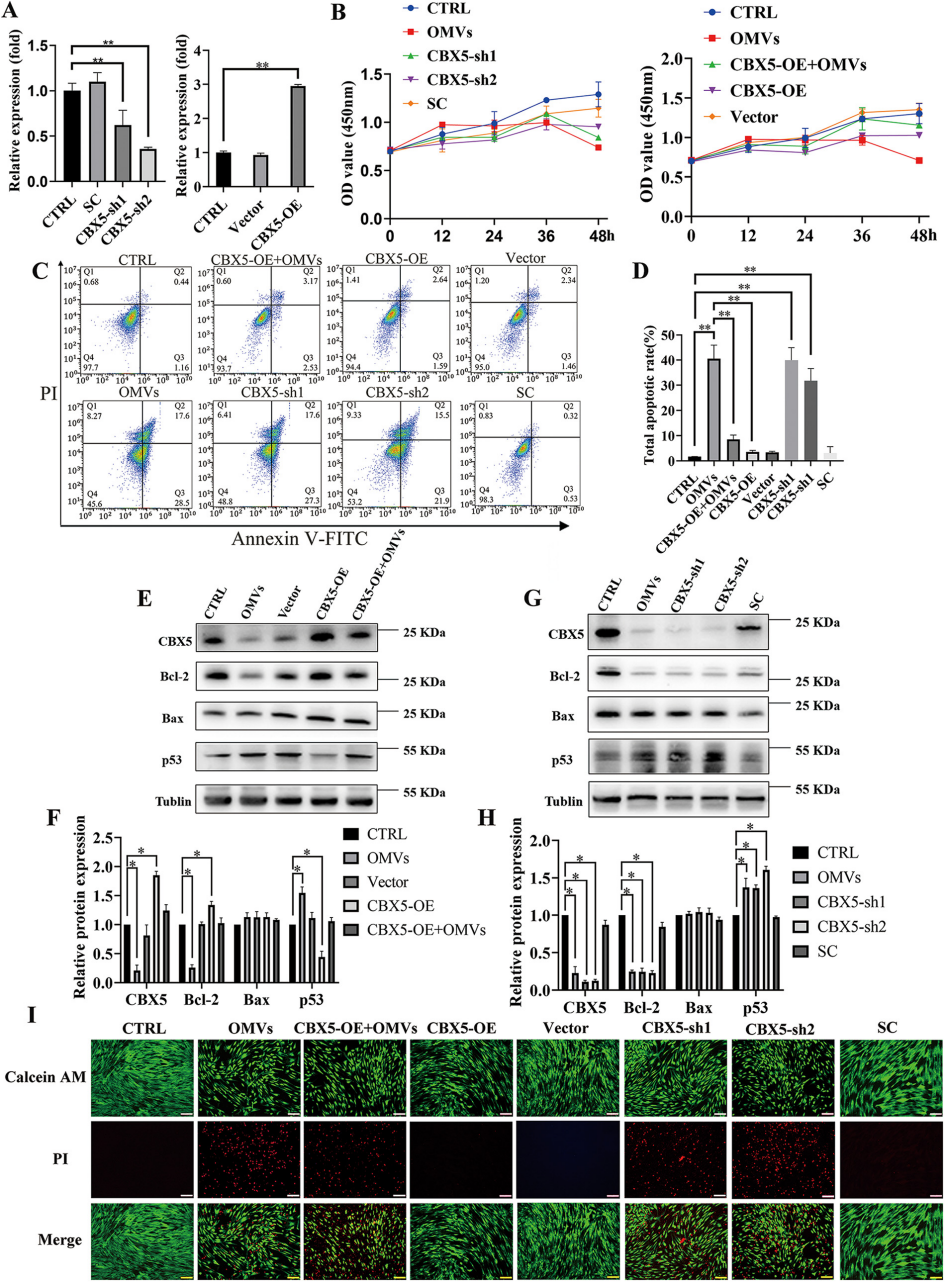
CBX5 regulates apoptosis via p53/Bcl-2 axis. (A) The efficacy of knockdown and overexpression of CBX5 was measured by qRT-PCR. (B) CCK-8 analysis of CBX5 knockdown and overexpression in hPDLCs; (C and D) flow cytometry analysis; (E to H) Western blot and the densitometric analyses of each group; (I) PI staining after overexpression and knockdown of CBX5. Data are shown as mean ± SD. Data between two groups were compared using Student's *t* test. Cell experiments were conducted three times independently. *, *P* < 0.05; **, *P* < 0.01.

To further understand the regulation of gene *CBX5* on apoptosis, hPDLCs were transfected with an plasmid overexpressing CBX5 (CBX5-OE) and a control vector, a CBX5 knockdown (CBX5-sh) plasmid, and a scramble knockdown plasmid (SC). The overexpressed and knockdown efficiency of CBX5 in hPDLCs was validated by qRT-PCR and Western blotting (Fig. 6A and E to H). CCK-8 showed that cell viability was reduced in the CBX5-sh group compared with those in the control and SC groups and was enhanced in the CBX5-OE+OMVs group compared with that in the OMVs group after transfections in hPDLCs. Furthermore, downregulated CBX5 significantly decreased cell viability compared to those in the control and SC groups, while upregulated CBX5 reversed the inhibitory influence of P. gingivalis OMVs (Fig. 6B). Additionally, downregulated CBX5 significantly increased cell apoptosis, consistent with the OMVs group, and overexpressed CBX5 rescued the promotional effect of P. gingivalis OMVs on cell apoptosis in hPDLCs, as shown by PI staining and flow cytometric analysis (Fig. 6C, D, and I). More importantly, in the CBX5-sh plasmid-transfected cells, p53 showed a significant increase in expression, while Bcl-2 showed a significant downregulation, displaying a similar pattern to the P. gingivalis OMV-stimulated group. While overexpressed CBX5 rescued the increased expression of p53, Bcl-2 was increased (Fig. 6E to H). As expected, PUMA and NOXA showed similar changes in p53 expression (Fig. S4E to H).

### p53 methylation suppressed by P. gingivalis OMVs in hPDLCs via CBX5.

**FIG 7:**
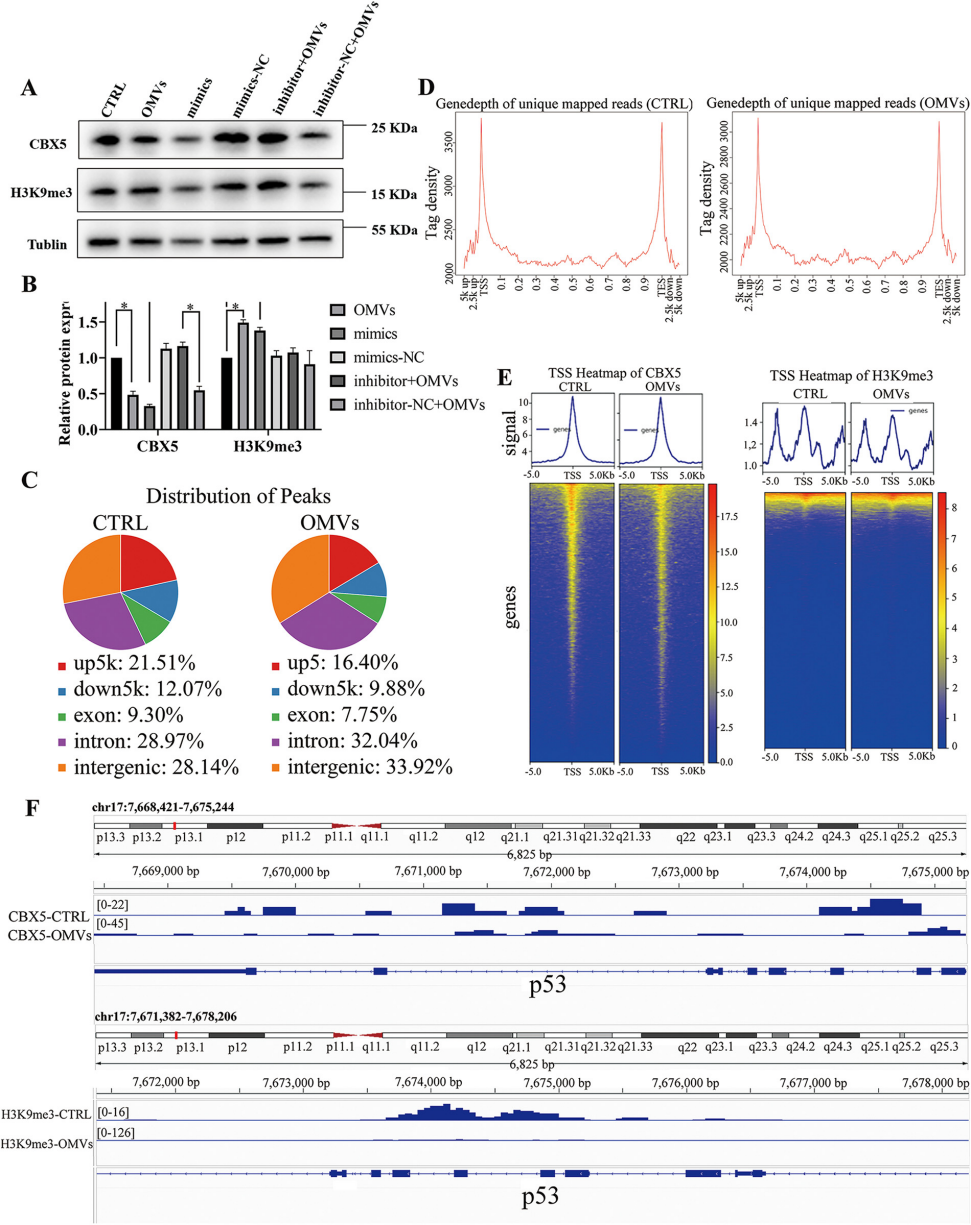
CBX5 regulates apoptosis through methylation in P. gingivalis OMV-stimulated hPDLCs. (A and B) The methylation level was measured by Western blotting. (C and D) The CUT&Tag technique was employed. Peak calling analysis of hPDLCs treated with or without P. gingivalis OMVs was performed. (E) TSS heat map of CBX5 and H3K9me3; (F) Iintegrative Genomics Viewer showing the signals of CBX5 and H3K9me3 at p53 loci. *, *P* < 0.05.

**FIG 8:**
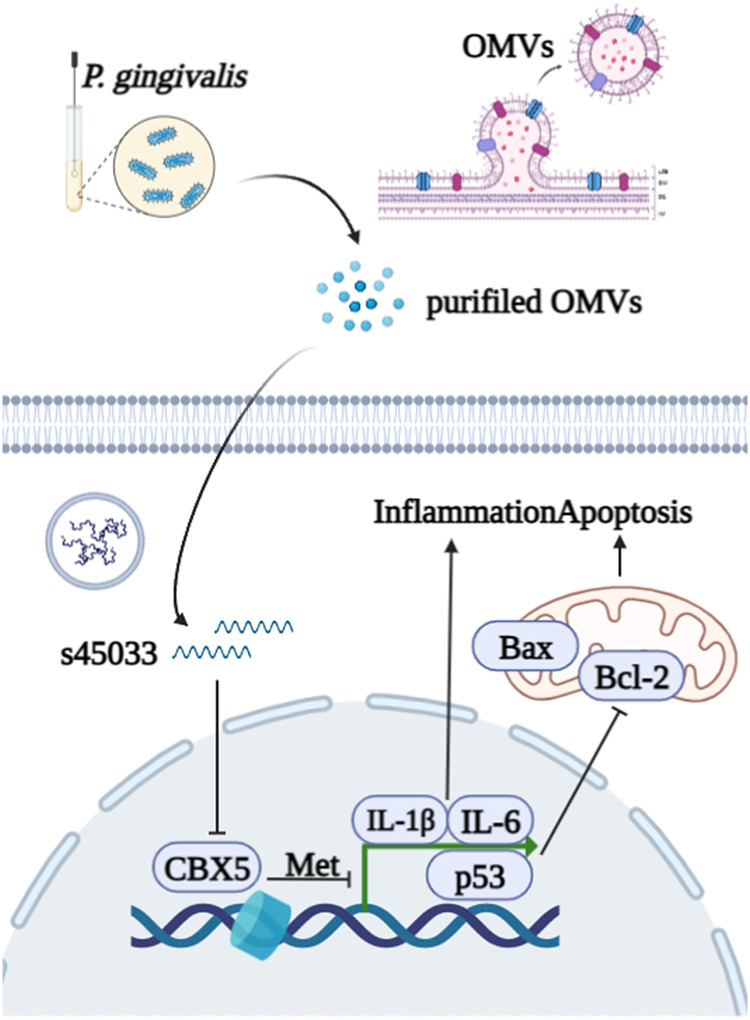
Schematic diagram of sRNA45033 packaged by P. gingivalis OMVs suppressing CBX5 to regulate apoptosis through p53 in hPDLCs.

To further understand the mechanism of how OMVs control p53 expression, we hypothesized that CBX5 regulates the epigenetic remodeling (H3K9me3, in this case) of the p53 gene, which results in the change in expression. Western blotting confirmed that decreased CBX5 reduced the level of H3K9me3 in hPDLCs (Fig. 7A and B). CUT&Tag (cleavage under targets and tagmentation) analysis was conducted to elucidate the connection between CBX5 and p53. Following the selection of hPDLCs with P. gingivalis OMVs, peak calling and transcriptional start site (TSS) profile analysis showed that CBX5 bound to the p53 gene similarly (Fig. 7C and D). For hPDLCs treated with P. gingivalis OMVs, the signal intensity was clearly lower than in those treated without OMVs (Fig. 7E). Consistently, the signal intensity of H3K9me3 of the p53 gene treated with P. gingivalis OMVs was lower than in those treated without OMVs (Fig. 7F). These results suggested that P. gingivalis OMVs reduced the level of CBX5 to regulate the methylation of p53, which determines the fate of hPDLCs (Fig. 8).

## DISCUSSION

The Gram-negative anaerobic bacterium P. gingivalis has been considered the keystone pathogen in periodontitis (6). Recent findings have suggested P. gingivalis plays an essential role in systemic diseases, such as cardiovascular diseases, rheumatoid arthritis, diabetes, pregnancy problems, Alzheimer’s disease, and insulin resistance (35–38). Henceforth, it is imperative to form a deeper understanding of the mechanism of P. gingivalis in periodontitis.

OMVs produced by periodontal bacteria have been demonstrated to take part in the progress of periodontal inflammation and tissue destruction (39). Periodontal pathogen OMVs are also related to distant organ dysfunction, including cardiovascular, brain, and joint dysfunction (40–42). Despite the explored effects in the periodontium, in-depth knowledge of the roles of these vesicles would significantly contribute to understanding the development of periodontitis and its related systemic diseases (43, 44).

Previous research had found that P. gingivalis was the most prolific OMV producer among the most proportionally increased bacteria during periodontal disease (45). P. gingivalis OMVs are actively involved in the progress of periodontal inflammation, immunomodulation of the host, and tissue destruction (46, 47). The average diameter of OMVs is 202.8 ± 71.87 nm, which was consistent with the range of the former study (11, 48, 49). The different average diameters could be result from OMVs’ fusion after ultracentrifugation or osmotic pressure after elution. The size difference is worth exploring because there is possibility that the size of OMVs could determine the biofunction of OMVs (50). The nanosized P. gingivalis OMVs could invade cells, as we confirmed in the endocytosis assay that hPDLCs successfully took up isolated P. gingivalis OMVs. Both silver staining and Coomassie blue staining showed that the protein bands were similar between P. gingivalis and *P. gingvalis* OMVs. However, the different distributions of protein content suggested their different functions in host-pathogen interaction. The isolated P. gingivalis OMVs were confirmed to promote bone resorption *in vivo*. Transcriptome sequencing (RNA-seq) was applied to compare the different functions between P. gingivalis OMVs and LPS. The annotated pathways of P. gingivalis OMVs were related to gene regulation, mRNA processing, and cell cycle process, while LPS was involved in inflammatory pathways. To investigate the detailed biofunction of isolated P. gingivalis OMVs, OMV-administered hPDLCs were found to be less proliferative and reduced viability. Generally, the decrease of mitochondrial membrane potential is considered to be one of the indicators of mitochondrial dysfunction and an early feature of apoptotic cells. The irregulated MMP also indicated aberrant reactive oxygen species (ROS) levels and showed signs of early apoptosis stimulated by P. gingivalis OMVs. NLRP3 and the proinflammatory cytokines IL-1β and IL-6 were much more elevated, which was consistent with other studies (51). TNF-α was also evaluated, however, with few changes: this was probably caused by the complex content within the P. gingivalis OMVs. Choi and colleagues found that only IL-5, IL-13, and IL-15 were changed in Jurkat T cells after transfections with synthetic msRNAs (17). Thus, future study of the relationship between P. gingivalis OMVs and proinflammatory cytokines will also help to clarify the roles of P. gingivalis OMVs in the pathogenesis of periodontitis. The apoptosis level was significantly higher when hPDLCs were treated with P. gingivalis OMVs, and the expression changes of apoptosis-related proteins p53, Bcl-2, and Bax indicated a connection between P. gingivalis OMVs and the apoptosis pathway.

OMVs contain genetic material that has been suggested as a novel gene transfer system (16). We hypothesized that msRNAs within the P. gingivalis OMVs regulate host genes in a way like miRNA, which binds to target mRNA. We selected the 10 highest predicted msRNAs from former sequencing results to identify the msRNA regulating apoptosis in hPDLCs. Five more highly expressed msRNAs in the isolated P. gingivalis OMVs were then verified and selected to analyze their downstream targets, and CBX5 was screened out as a possible candidate.

CBX5 is an important architectural protein that forms heterochromatin, which is compact in structure and transcriptionally repressed. CBX5 induces epigenetic remodeling like H3K9 methylation in the promoter region and silences the adjacent gene (52). The expression level of CBX5 was confirmed to be significantly reduced after OMV administration. To understand the mechanism of this regulation, the binding of sRNA45033 and CBX5 3′ UTR was confirmed by a dual-luciferase reporter assay. The sRNA45033 mimic and inhibitor confirmed that sRNA45033 inhibited cell proliferation and regulated apoptosis via CBX5 expression.

p53 induces apoptosis through upregulation of proapoptotic BH3-only members of the Bcl-2 protein family (such as BIM, NOXA, and PUMA), which bind and inhibit prosurvival Bcl-2 proteins, in turn, releasing proapoptotic Bcl-2 family members like Bax (53). Studies have suggested that CBX5 was (i) involved in inflammatory response-related apoptosis genes, (ii) repressed apoptosis in renal cell carcinoma, (iii) interacted with p53 in several cancer cell lines, and (iv) and was expressed to a higher level with p53 loss in some cell lines (31, 54–56). These prompted us to investigate the underlying mechanism of CBX5 regulating apoptosis. Indeed, overexpression and knockdown of CBX5 further proved that CBX5 is a key regulator between sRNA45033 and p53/Bcl-2/Bax.

CUT&Tag is a novel technique for mapping chromatin features and understanding epigenetic regulation in the genome. In short, a specific antibody binds to a chromatin protein and then tethers with a protein A-Tn*5* transposase fusion protein. Upon activation, this protein generates sequencing libraries with remarkable signal-to-noise ratio and reliability (57, 58). To explore the underlying mechanism, CUT&Tag analysis and Western blotting were carried out, and we found that CBX5 controlled the H3K9me3 level of p53, which forms heterochromatin to affect p53 expression. Subsequently, Bcl-2 was downregulated by the elevated p53 expression and the host cells underwent apoptosis.

This study uncovered a novel complex regulatory system of P. gingivalis OMVs; however, there are some limitations to this study. First, P. gingivalis OMVs contain many other msRNAs that also possibly contribute to the progress of periodontitis. Second, we verified only the p53/Bcl-2/Bax axis, yet apoptosis regulation is an extremely complicated network. The expression of Bax was only slightly changed after the administration of OMVs; this might be caused by the internal high level of Bax in hPDLCs. Third, to further confirm the methylation of the p53 and involvement of CBX5, chromatin immunoprecipitation will be utilized in the future. Fourth, more detailed *in vivo* experiments are needed to provide solid evidence on the roles of P. gingivalis OMVs and identify their effectiveness as possible treatment targets. Due to these limitations, we will further explore the role and function of P. gingivalis OMVs systemically. A more in-depth understanding of P. gingivalis OMVs periodontitis will also be beneficial in the aspect of treating periodontitis.

In summary, our data have suggested a novel mechanism in which sRNA45033 packaged by P. gingivalis OMVs inhibits CBX5 expression in host cells, which lowers the H3K9me3 level of p53. Accordingly, p53 expression was elevated and Bcl-2, the key protein in apoptosis, was downregulated and ultimately promoted apoptosis in the host cells.

## MATERIALS AND METHODS

### Bacterial cultures.

P. gingivalis ATCC 33277 was purchased from the Shanghai Biology Collection Center (Shanghai, China), and the culture method was adopted from previously studies (17, 59). Briefly, cells were cultured in brain heart infusion broth (Oxoid, Basingstoke, United Kingdom) containing 10% sterile defibrinated sheep blood, 0.5% hemin (Hopebiol, Shandong, China), and 0.1% vitamin K_1_ (Hopebiol, Shandong, China) in an anaerobic chamber (80% N_2_, 10% O_2_, 10% H_2_) at 37°C. Bacteria were harvested after growing for 48 h under anaerobic conditions in an anaerobic chamber.

### Isolation and identification of P. gingivalis OMVs.

The isolation of P. gingivalis OMVs followed established protocols (47, 60). Briefly, for freshly grown P. gingivalis cells (optical density at 600 nm [OD_600_] of 1.0, equivalent to 9 × 10^9^ CFU), the bacterial culture medium was centrifuged at 8,000 × *g* for 15 min at 4°C to remove the bacterial cells. The supernatant was collected and filtered using a 0.22-μm-pore syringe filter (Millipore, MA, USA). The samples were centrifuged at 100,000 × *g* for 2 h at 4°C (Fig. 1A). The OMV fractions were dissolved in 200 μL of phosphate-buffered saline (PBS) and stored at −80°C until needed.

OMVs were analyzed by transmission electron microscopy (TEM). The OMV samples were applied to 200-mesh Formvar-carbon grids (Ted Pella) with 2% phosphotungstic acid (Solarbio, Beijing, China). Samples were dried on the grids, viewed, and photographed with a Hitachi model H-7500 transmission electron microscope (Hitachi, Tokyo, Japan). The diameter of P. gingivalis OMVs was measured using dynamic light scattering (Malvern Zetasizer Nano ZS90; Malvern, United Kingdom).

For silver staining, concentrations of protein were measured using a bicinchoninic acid (BCA) protein assay kit (Beyotime, Shanghai, China). P. gingivalis OMVs (400 ng of total protein) and an equal amount of P. gingivalis proteins were separated by SDS-PAGE. The proteins on SDS gels were visualized by using Silver Stain Plus (Beyotime Biotechnology, Nanjing, China).

### Animals.

The animal study was reviewed and approved by Institutional Animal Care and Use Committee of Nanjing Medical University (IACUC-2006020). SD rats (male, 6 to 7 weeks old) were purchased from the Animal Core Facility of Nanjing Medical University. They were caged under specific-pathogen-free (SPF) conditions, fed a normal diet, and subjected to constant temperature and humidity for 12 h each of alternating light/darkness cycles. SD rats were randomly divided into four groups: (i) the control rats (CTRL group); (ii) healthy rats treated with P. gingivalis OMVs (OMVs group); (iii) the chronic periodontitis model rats (CP group), and (iv) chronic periodontitis rats treated with P. gingivalis OMVs (CP+OMVs group). The chronic periodontitis modeling method was conducted as follows. A standard ligation wire (0.25 mm) was passed through the proximal and distal adjacent spaces of the bilateral maxillary M1, ligated around the dental neck, placed in the gingival sulcus as far as possible, and knotted in the mesiobuccal side of M1 (45). The ligation wire was checked daily for shedding and was religated when the wire detached. Periodontal ligation was maintained for 4 weeks to keep periodontal tissue inflammation in a stable state to establish the model of periodontitis (46, 47). Two groups received P. gingivalis OMVs at a dose (20 μL) of 5 μg/μL three times per week by local injection. The other group of rats received a comparable volume of PBS (Fig. 2A). In the animal experiments, the investigator was blind to the group allocation. After 4 weeks, the rats were euthanized and the femurs were harvested for microcomputed tomography (micro-CT) and histological analysis.

### Micro-CT and histological analysis.

The rats were sacrificed 4 weeks after surgery, and the cranial tissue was collected. The tissues were fixed with 4% paraformaldehyde (PFA) for 1 week and 75% ethanol for micro-CT evaluation. 3D images of the mineralized tissues were reconstructed using Sky scan software. The bone volume/tissue volume (BV/TV) of each sample was collected for analysis. After micro-CT evaluation, the tissues were demineralized in 14% EDTA solution for 3 months, dehydrated by an automatic dehydrator, and embedded in paraffin. Paraffin sections were cut into tissue sections 5 mm thick for hematoxylin and eosin (H&E), TRAP staining, and immunochemistry.

### Cell culture.

The cell study was reviewed and approved by Ethics Committee of the Affiliated Stomatological Hospital of Nanjing Medical University (PJ2021-089-001). hPDLCs were chosen to conduct *in vitro* studies (61). hPDLCs were extracted from the middle third of the root surfaces of young patients (12 to 16 years old) requiring orthodontic treatment, and informed consent was obtained. The obtained hPDLCs were confirmed by the immunochemistry staining of keratin and vimentin (see Fig. S2 in the supplemental material). The cells were then cultured in α minimum essential medium (α-MEM) supplemented with 15% fetal bovine serum (FBS) (Gibco, CA, USA) and 1% streptomycin-penicillin (Gibco, CA, USA) and incubated at 37°C in a humidified atmosphere with 5% CO_2_. Upon attaining 90% confluence, hPDLCs were maintained in α-MEM with 10% FBS and used between passages 3 and 6.

### Endocytosis analysis.

Dil (1,1′-dioctadecyl-3,3,3,3′-tetramethylindocarbocyanine perchlorate) was used to label the P. gingivalis OMVs’ lipid membrane. The isolated P. gingivalis OMVs were incubated with 2 μL of Dil (Molecular Probes, CA, USA) in 100 μL PBS for 15 min. Subsequently, the Dil-labeled P. gingivalis OMVs were harvested and incubated with hPDLCs at 37°C for 6 h. Then, the cells were fixed with 4% paraformaldehyde for 20 min and stained with phalloidin and DAPI (4′,6-diamidino-2-phenylindole) (Beyotime Biotechnology, Shanghai, China). Fluorescence images were captured under a confocal microscope (Leica, Heidelberg, Germany).

### Cell Counting Kit-8 analysis.

To investigate the effects of P. gingivalis OMVs (10 μg/mL) on the growth of cultured cells, a medium containing 1 × 10^5^ hPDLCs was pipetted into 96-well tissue culture plates. The cells were allowed to attach and grow for 48 h. After the indicated treatments, the cells were washed with PBS buffer three times, followed by the addition of 100 μL α-MEM medium supplemented with 10% Cell Counting Kit-8 (CCK-8), and then the cells were incubated at 37°C for 2 h. The optical density at 450 nm (OD_450_) was measured by a microplate reader (SpectraMax190; Molecular Devices, San Jose, CA, USA). At least three separate experiments were done for statistical analysis.

### Calcein/propidium iodide staining.

Medium containing 2 × 10^4^ hPDLCs was pipetted into 24-well plates, and the cells were allowed to attach and grow for 48 h. After the indicated treatments, the cells were washed with PBS buffer three times and then were analyzed with the LIVE/DEAD viability/cytotoxicity assay kit for animal cells (KeyGEN BioTECH, Jiangsu, China), with Calcein AM and PI for 30 min, which could stain the cells to distinguish the living cells (green) from the dead ones (red). The samples were then observed under a fluorescence microscope (DMI6000B; Leica, Heidelberg, Germany).

### JC-1 staining for mitochondrial membrane potential.

Mitochondrial membrane potential was measured using the mitochondrial membrane potential probe JC-1 staining dye in hPDLCs. Briefly, cells were cultured in a glass bottom dish with or without pretreatment with P. gingivalis OMVs (10 μg/mL) for 24 h. After JC-1 working solution was added, the cells were maintained in a CO_2_ incubator for 20 min. The staining solution was removed, and then the cells were gently washed twice with JC-1 staining buffer. Fluorescence was detected by confocal microscopy (Leica, Heidelberg, Germany).

### Quantitative reverse transcription-PCR analysis of sRNAs and mRNAs.

Total RNA was isolated by the RNA Simple Total kit (TianGen, Beijing, China), and P. gingivalis OMV sRNAs were isolated by the RNAeasy small RNA isolation kit (Beyotime Biotechnology, Shanghai, China). In addition, cDNA was generated with a PrimeScript RT master mix for quantitative PCR (qPCR) (TaKaRa, Kyoto, Japan), followed by analysis using an SYBR Premix *Ex Taq* II (TaKaRa, Kyoto, Japan). Stem-loop reverse transcription was utilized to reverse msRNAs because of the short fragment. PCR conditions consisted of an initial 30 s of denaturation at 95°C, followed by 40 cycles of 95°C for 5 s, and 60°C for 30 s. All reactions were performed in triplicate. Change in transcript abundance of all tested genes was calculated using the threshold cycle (2^−ΔΔ^*^CT^*) method. The primers used in the study are listed in Table 1 and Table 2.

**TABLE 1:** Primers for msRNA screening

Primer	Sequence
sRNA45033-RT	GTCGTATCCAGTGCGTGTCGTGGAGTCGGCAATTGCACTGGATACGACCAGCCCT
sRNA45033-F	AGCGAGGGAAACCAATAGCAG
sRNA38803-RT	GTCGTATCCAGTGCGTGTCGTGGAGTCGGCAATTGCACTGGATACGACGGCCGTA
sRNA38803-F	AGGTAAGGGACAGGGACAGA
sRNA4378-RT	GTCGTATCCAGTGCGTGTCGTGGAGTCGGCAATTGCACTGGATACGACTAACCCA
sRNA4378-F	TCTGAGGGTGGGATTATGAGCTA
sRNA29795-RT	GTCGTATCCAGTGCGTGTCGTGGAGTCGGCAATTGCACTGGATACGACGGGTTC
sRNA29795-F	GCTGAGGGTGGGAAATGAAGT
sRNA16418-RT	GTCGTATCCAGTGCGTGTCGTGGAGTCGGCAATTGCACTGGATACGACCTGGCAA
sRNA16418-F	GTGGGTTACACCGGACCTC
sRNA43507-RT	GTCGTATCCAGTGCGTGTCGTGGAGTCGGCAATTGCACTGGATACGACGCCAGAG
sRNA43507-F	TGGGATCAGTTGGTTGGAAAGA
sRNA23392-RT	GTCGTATCCAGTGCGTGTCGTGGAGTCGGCAATTGCACTGGATACGACTGTCACC
sRNA23392-F	CTGGTGGGATAAAGCGAGAGG
sRNA30540-RT	GTCGTATCCAGTGCGTGTCGTGGAGTCGGCAATTGCACTGGATACGACGCTGACA
sRNA30540-F	GGGTTGCAGGACGCGAT
sRNA13572-RT	GTCGTATCCAGTGCGTGTCGTGGAGTCGGCAATTGCACTGGATACGACGCAGAG
sRNA13572-F	GCGAGGTGGGATAAGGAAGC
sRNA16110-RT	GTCGTATCCAGTGCGTGTCGTGGAGTCGGCAATTGCACTGGATACGACGTAGCAG
sRNA16110-F	GCGTGGCATTGGTATTGTTGG
P. gingivalis 16S RNA-F	TGTAGATGACTGATGGTGAAAACC
P. gingivalis 16S RNA-R	ACGTCATCCACACCTTCCTC
sRNA-R	CAGTGCGTGTCGTGGAGT
GAPDH-F	CTGGGCTACACTGAGCACC
GAPDH-R	AAGTGGTCGTTGAGGGCAATG
β-Actin-F	GTCCCTCACCCTCCCAAAAG
β-Actin-R	GCTGCCTCAACACCTCAACCC

**TABLE 2:** Primers for qRT-PCR

Primer	Gene	Sequence	
		Forward	Reverse
s43507	ENST00000321801(BOLL)	TGCCTTTGAATAACCCAACAAGT	TTCACAGACCCATACTGGGAA
	ENST00000611864(MGAT4C)	TCACCTATCGCTACCTAGCTG	GGCATCACGCCAGGAAGAAT
	ENST00000261427(UBE2K)	GTTCCGTCACAGGGGCTATTT	AATACCGTGCGGAGAGTCATT
	ENST00000321662(GPR137C)	ACCTGGCGGAGGTTATATGTAA	TCTCCATGAACTAGCATTGCG
	ENST00000410080(PRPF40A)	AGAGAGCGAATATGCCTCCTG	GCATTACTGACGACATCATTCCA

s16418	ENST00000303177(HMP19)	GCCGCCTTCAGTTGAGGAT	TCCGGCTGATATTCCGTTCTT
	ENST00000244745(SOX4)	GCCGAGTGGAAACTTTTGTCG	GGCAGCGTGTACTTATCCTTCT
	ENST00000260197(SORL1)	CAAGGTGTACGGACAGGTTAGT	CCAATGCCAGGCTATCTCG
	ENST00000370598(ADGRB3)	TGCCCAAGACTTCTGGTGTTC	AAGTTAGAGCAGCTAAGGTCCT
	ENST00000261798(CSNK1A1)	AGTGGCAGTGAAGCTAGAATCT	CGCCCAATACCCATTAGGAAGTT

s30540	ENST00000615466(ZNF189)	AGCCTTTCGATTAAGCACATACC	AGCTCCGACTGAAACTTTTTCC
	ENST00000266643(MARCH9)	ATCTCCCTGACGGTCATCGAG	GGCTGAAGGGCTGAGTGAG
	ENST00000370689(PRKACB)	CCATGCACGGTTCTATGCAG	GTCTGTGACCTGGATATAGCCTT
	ENST00000320848(MRFAP1L1)	CCCCTGGACATAGACGAGGT	CCGTGCTCGCGTATAAGAGAC
	ENST00000356450(NUDT10)	CCAGACACGGACCTACGA	CCTTGACTCCCGCCTCTT

s23392	ENST00000379565(RPS6KA3)	CGCTGAGAATGGACAGCAAAT	TCCAAATGATCCCTGCCCTAAT
	ENST00000614987(RPS6KA5)	CTCCTCACTGTCAAGCACGAG	GCCTTTTGAACGATTGTTGCCT
	ENST00000011619(RANBP9)	GGCCGTGGACGAACAAGAG	GAACTGACGCGGCATCTTTT
	ENST00000234453(PLEKHA3)	ACTGTGACCTCTTAATGCAGC	CTCAAGCGTTGTGATGAATGTG
	ENST00000491143(ONECUT2)	GGAATCCAAAACCGTGGAGTAA	CTCTTTGCGTTTGCACGCTG

s38803	ENST00000542274(CDH3)	ATCATCGTGACCGACCAGAAT	GACTCCCTCTAAGACACTCCC
	ENST00000614512(SATB2)	CATGCCACAGTCCGCAATG	GGCCCAGAACACAATAGTCTGA
	ENST00000338965(NCR3LG1)	TGTGAGTCAAGTGGGTTCTACC	CATGCCGTACCACACACTG
	ENST00000285737(LONP2)	ATGCTGTGAGCCTAGAGGAG	GCTCTGGCATACTAGATGTTCG
	ENST00000267890(TTBK2)	CAATCAACGCACATCGGAACA	GAGCCTACTTGCTCCTTGTCC

s45033	ENST00000209875(CBX5)	AACAGTGCCGATGACATCAAA	GCCCCAATGATCTTTTCTGGT
	ENST00000335420(KLHDC10)	TACGATGGGACCCAGTTAGGA	TGTGGCCTCTCAAAAACCTGT
	ENST00000358127(PAX5)	AAACCAAAGGTCGCCACAC	GTTGATGGAACTGACGCTAGG
	ENST00000333137(SMTN)	GGGATCGTGTCCACAAGTTCA	GCTACTCCTCGTTGCTCCTT
	ENST00000548729(POC1B-GALNT4)	AATACTATGCCTCCCTTTG	AGCCCACTTTCAGTTTCA

### RNA library construction and sequencing.

A total amount of 1 μg RNA per sample was used as input material for the RNA sample preparations. Sequencing libraries were generated using NEBNext Ultra RNA library prep kit for Illumina (NEB, MA, USA) following the manufacturer’s recommendations, and index codes were added to attribute sequences to each sample. Briefly, mRNA was purified from total RNA using poly(T) oligonucleotide-attached magnetic beads. Fragmentation was carried out using divalent cations under elevated temperature in NEBNext first-strand synthesis reaction buffer (5×). First-strand cDNA was synthesized using random hexamer primer and Moloney murine leukemia virus (MMuLV) reverse transcriptase (RNase H^−^). Second-strand cDNA synthesis was subsequently performed using DNA polymerase I and RNase H. The remaining overhangs were converted into blunt ends via exonuclease/polymerase activities. After adenylation of 3′ ends of DNA fragments, NEBNext adaptor with a hairpin loop structure was ligated to prepare for hybridization. To select cDNA fragments preferentially 250 to ~300 bp in length, the library fragments were purified with the AMPure XP system (Beckman Coulter, CA, USA). Then, 3 μL USER enzyme (NEB, MA, USA) was used with size-selected, adaptor-ligated cDNA at 37°C for 15 min, followed by 5 min at 95°C before PCR. Then, PCR was performed with Phusion high-fidelity DNA polymerase, universal PCR primers, and index (X) primer. Finally, PCR products were purified (AMPure XP system) and library quality was assessed on the Agilent Bioanalyzer 2100 system.

The clustering of the index-coded samples was performed on a cBot cluster generation system using TruSeq PE cluster kit v.3-cBot-HS (Illumia, NEB, MA, USA) according to the manufacturer’s instructions. After cluster generation, the library preparations were sequenced on an Illumina Novaseq platform and 150-bp paired-end reads were generated.

### Western blot.

After being washed with PBS, hPDLCs were lysed in lysis buffer (whole-cell lysis assay; KeyGEN BioTECH, Jiangsu, China). Concentrations of protein were measured using a BCA protein assay kit. Proteins were separated on SDS-polyacrylamide gels and transferred to polyvinylidene difluoride (PVDF) membranes (Millipore, Bedford, MA, USA). Membranes were incubated with primary antibodies to Bcl-2 (1:1,000) (Abcam, Cambridge, United Kingdom), Bax (1:1,000) (Abcam, Cambridge, United Kingdom), p53 (1:1,000) (Proteintech, Chicago, IL, USA), PUMA (1:1,000) (Proteintech, Chicago, IL, USA), NOXA (1:1,000) (Affinity Biosciences, Cincinnati, OH, USA), CBX5 (1:500) (Proteintech, Chicago, IL, USA), NLRP3 (1:1,000) (Abcam, Cambridge, United Kingdom), H3K9me3 (1:1,000) (Abcam, Cambridge, United Kingdom), and tubulin (1:1,000) (Proteintech, Chicago, IL, USA) overnight at 4°C. Subsequently, membranes were then incubated with corresponding secondary antibodies (Proteintech, Chicago, IL, USA) at room temperature for 60 min. Blotted bands were visualized after ECL enhanced chemiluminescence exposure by a chemiluminescent gel imaging system (Tanon, Shanghai, China). The protein levels were analyzed using ImageJ software; at least three biological replicates were included.

### Flow cytometry.

We analyzed the apoptosis of hPDLCs using flow cytometry. After the indicated treatments, for the cell apoptosis analysis, hPDLCs were collected separately and incubated with the Alexa Fluor 488-annexin V/dead cell apoptosis kit (Invitrogen, CA, USA), using annexin V-fluorescein isothiocyanate-propidium iodide, and the cells were incubated at room temperature for 15 min. Subsequently, the apoptotic cells were analyzed using a FACSCalibur (Becton Dickenson, Franklin Lakes, NJ, USA).

### ELISA.

Il-1β, TNF-α, and IL-6 in the cell supernatant were determined via an enzyme-linked immunosorbent assay (ELISA) by using commercially available ELISA sets (Neobiscience, Shenzhen, China) following the instructions of the manufacturer. All samples were measured in duplicate.

### Caspase-3 activity assay.

Activities of caspase-3 were measured using GreenNuc caspase-3 assay kit for live cells (Beyotime, Shanghai, China). In brief, cells were cultured in 96-well black plates with or without pretreatment with P. gingivalis OMVs (10 μg/mL) for 24 h, and the untreated cells were used as the negative control. The inhibitor group added a certain concentration of inhibitor (5 μM). The cells were replaced with fresh culture medium containing substrate (5 μM) or PBS. The Ac-DEVD-CHO inhibitor group was supplemented with the original concentration of inhibitor (5 μM). Cells were incubated at room temperature in dark for 15 to 30 min (longer if necessary). The microplate reader (SpectraMax 190; Molecular Devices) was set with an excitation wavelength of 485 nm and an emission wavelength of 515 nm. For adherent cells, bottom reading was recommended.

### Dual-luciferase reporter assay.

pEZX-FR02 luciferase reporter vector (GeneCopoeia, MD, USA) containing a 3′-UTR fragment of CBX5 with the predicted binding site or a mutant variant was prepared based on previous research (11). Luciferase activities were measured 48 h after transfection using the dual-luciferase reporter assay kit (Vazyme, Jiangsu, China). Firefly luciferase activity was normalized to *Renilla* luciferase activity for each sample. sRNA45033 mimic and inhibitor (reverse complement to mimic) were synthesized by Ribobio (Guangzhou, China).

### CUT&Tag analysis, library construction, and DNA sequencing.

The CUT&Tag library was prepared using the Hyperactive Universal CUT&Tag assay kit for Illumina (Vazyme, Jiangsu, China) according to the manufacturer’s instructions. A total of 100,000 cells were harvested, washed, and mixed with activated concanavalin A-coated magnetic beads at room temperature for 10 min. The mixture was resuspended in 50 μL antibody buffer consisting of primary antibody (1:50 dilution) and incubated overnight at 4°C. After being washed, 100 μL of pA/G-Tnp adapter complex (~0.04 μM) was added to the cells and incubated at room temperature for about 1 h. After washing, the cells were resuspended in Tagmentation buffer (50 μL) and incubated at 37°C for 1 h. Then, proteinase K treatment and DNA extract beads (Vazyme, Jiangsu, China) were used at 55°C for 10 min, and DNA was eluted. For PCR, the following thermocycler program was used: 72°C for 3 min, followed by 95°C for 3 min, 10 cycles of 98°C for 10 s and 60°C for 5 s, final extension at 72°C for 1 min, and hold at 4°C. Pooled libraries were purified with VAHTS DNA clean beads (Vazyme, Jiangsu, China). Sequencing was performed with an Illumina NovaSeq 6000 platform (provided by Annoroad Company, Beijing, China), with a sequencing depth of 6 Gb for each sample. The data were visualized using Integrative Genomics Viewer and Vazyme cloud services.

### Statistical analysis.

The results are expressed as means ± standard deviation (SD). Experiments were repeated independently at least three times. Statistical significance was assessed with Student's *t* test or analysis of variance (ANOVA) using SPSS software and GraphPad Prism 5. A *P* value of *<*0.05 was considered statistically significant.

### Data availability.

Data sets have been uploaded to the Gene Expression Omnibus under GEO accession no. GSE218606.

## Supplementary Material

Reviewer comments

## References

[1] Papapanou PN, Susin C. 2017. Periodontitis epidemiology: is periodontitis under-recognized, over-diagnosed, or both? Periodontol 2000 75:45–51. doi:10.1111/prd.12200. 28758302

[2] Kinane DF. 2001. Causation and pathogenesis of periodontal disease. Periodontol 2000 25:8–20. doi:10.1034/j.1600-0757.2001.22250102.x. 11155179

[3] Cheng R, Hu T, Bhowmick NA. 2015. Be resistant to apoptosis: a host factor from gingival fibroblasts. Cell Death Dis 6:e2009. doi:10.1038/cddis.2015.350. 26633715 PMC4720885

[4] Cecil JD, Sirisaengtaksin N, O’Brien-Simpson NM, Krachler AM. 2019. Outer membrane vesicle-host cell interactions. Microbiol Spectr 7. doi:10.1128/microbiolspec.PSIB-0001-2018. PMC6352913 30681067

[5] Lamkanfi M, Dixit VM. 2012. Inflammasomes and their roles in health and disease. Annu Rev Cell Dev Biol 28:137–161. doi:10.1146/annurev-cellbio-101011-155745. 22974247

[6] Hajishengallis G, Darveau RP, Curtis MA. 2012. The keystone-pathogen hypothesis. Nat Rev Microbiol 10:717–725. doi:10.1038/nrmicro2873. 22941505 PMC3498498

[7] He Z, Jiang W, Jiang Y, Dong J, Song Z, Xu J, Zhou W. 2022. Anti-biofilm activities of coumarin as quorum sensing inhibitor for *Porphyromonas gingivalis*. J Oral Microbiol 14:2055523. doi:10.1080/20002297.2022.2055523. 35368854 PMC8967191

[8] Hajishengallis G, Lamont RJ. 2012. Beyond the red complex and into more complexity: the polymicrobial synergy and dysbiosis (PSD) model of periodontal disease etiology. Mol Oral Microbiol 27:409–419. doi:10.1111/j.2041-1014.2012.00663.x. 23134607 PMC3653317

[9] Jia L, Han N, Du J, Guo L, Luo Z, Liu Y. 2019. Pathogenesis of important virulence factors of *Porphyromonas gingivalis* via Toll-like receptors. Front Cell Infect Microbiol 9:262. doi:10.3389/fcimb.2019.00262. 31380305 PMC6657652

[10] Hajishengallis G, Lambris JD. 2011. Microbial manipulation of receptor crosstalk in innate immunity. Nat Rev Immunol 11:187–200. doi:10.1038/nri2918. 21350579 PMC3077082

[11] Liu D, Liu S, Liu J, Miao L, Zhang S, Pan Y. 2021. sRNA23392 packaged by *Porphyromonas gingivalis* outer membrane vesicles promotes oral squamous cell carcinomas migration and invasion by targeting desmocollin-2. Mol Oral Microbiol 36:182–191. doi:10.1111/omi.12334. 33764008

[12] Schwechheimer C, Kuehn MJ. 2015. Outer-membrane vesicles from Gram-negative bacteria: biogenesis and functions. Nat Rev Microbiol 13:605–619. doi:10.1038/nrmicro3525. 26373371 PMC5308417

[13] Ma L, Cao Z. 2021. Membrane vesicles from periodontal pathogens and their potential roles in periodontal disease and systemic illnesses. J Periodontal Res 56:646–655. doi:10.1111/jre.12884. 33826135

[14] Srisatjaluk R, Doyle RJ, Justus DE. 1999. Outer membrane vesicles of *Porphyromonas gingivalis* inhibit IFN-gamma-mediated MHC class II expression by human vascular endothelial cells. Microb Pathog 27:81–91. doi:10.1006/mpat.1999.0287. 10458919

[15] Qi M, Miyakawa H, Kuramitsu HK. 2003. *Porphyromonas gingivalis* induces murine macrophage foam cell formation. Microb Pathog 35:259–267. doi:10.1016/j.micpath.2003.07.002. 14580389

[16] Koeppen K, Hampton TH, Jarek M, Scharfe M, Gerber SA, Mielcarz DW, Demers EG, Dolben EL, Hammond JH, Hogan DA, Stanton BA. 2016. A novel mechanism of host-pathogen interaction through sRNA in bacterial outer membrane vesicles. PLoS Pathog 12:e1005672. doi:10.1371/journal.ppat.1005672. 27295279 PMC4905634

[17] Choi J-W, Kim S-C, Hong S-H, Lee H-J. 2017. Secretable small RNAs via outer membrane vesicles in periodontal pathogens. J Dent Res 96:458–466. doi:10.1177/0022034516685071. 28068479

[18] Furuse Y, Finethy R, Saka HA, Xet-Mull AM, Sisk DM, Smith KLJ, Lee S, Coers J, Valdivia RH, Tobin DM, Cullen BR. 2014. Search for microRNAs expressed by intracellular bacterial pathogens in infected mammalian cells. PLoS One 9:e106434. doi:10.1371/journal.pone.0106434. 25184567 PMC4153649

[19] Weiberg A, Wang M, Lin F-M, Zhao H, Zhang Z, Kaloshian I, Huang H-D, Jin H. 2013. Fungal small RNAs suppress plant immunity by hijacking host RNA interference pathways. Science 342:118–123. doi:10.1126/science.1239705. 24092744 PMC4096153

[20] Westermann AJ, Förstner KU, Amman F, Barquist L, Chao Y, Schulte LN, Müller L, Reinhardt R, Stadler PF, Vogel J. 2016. Dual RNA-seq unveils noncoding RNA functions in host-pathogen interactions. Nature 529:496–501. doi:10.1038/nature16547.. 26789254

[21] Lee H-J. 2019. Microbe-host communication by small RNAs in extracellular vesicles: vehicles for transkingdom RNA transportation. Int J Mol Sci 20:1487. doi:10.3390/ijms20061487. 30934547 PMC6472211

[22] Tsatsaronis JA, Franch-Arroyo S, Resch U, Charpentier E. 2018. Extracellular vesicle RNA: a universal mediator of microbial communication? Trends Microbiol 26:401–410. doi:10.1016/j.tim.2018.02.009. 29548832

[23] Massé E, Escorcia FE, Gottesman S. 2003. Coupled degradation of a small regulatory RNA and its mRNA targets in Escherichia coli. Genes Dev 17:2374–2383. doi:10.1101/gad.1127103. 12975324 PMC218075

[24] Lee H-J, Hong S-H. 2012. Analysis of microRNA-size, small RNAs in Streptococcus mutans by deep sequencing. FEMS Microbiol Lett 326:131–136. doi:10.1111/j.1574-6968.2011.02441.x. 22092283

[25] Kang S-M, Choi J-W, Lee Y, Hong S-H, Lee H-J. 2013. Identification of microRNA-size, small RNAs in Escherichia coli. Curr Microbiol 67:609–613. doi:10.1007/s00284-013-0411-9. 23783561

[26] Han E-C, Choi S-Y, Lee Y, Park J-W, Hong S-H, Lee H-J. 2019. Extracellular RNAs in periodontopathogenic outer membrane vesicles promote TNF-α production in human macrophages and cross the blood-brain barrier in mice. FASEB J 33:13412–13422. doi:10.1096/fj.201901575R. 31545910 PMC6894046

[27] Ahmadi Badi S, Bruno SP, Moshiri A, Tarashi S, Siadat SD, Masotti A. 2020. Small RNAs in outer membrane vesicles and their function in host-microbe interactions. Front Microbiol 11:1209. doi:10.3389/fmicb.2020.01209. 32670219 PMC7327240

[28] Bannister AJ, Zegerman P, Partridge JF, Miska EA, Thomas JO, Allshire RC, Kouzarides T. 2001. Selective recognition of methylated lysine 9 on histone H3 by the HP1 chromo domain. Nature 410:120–124. doi:10.1038/35065138. 11242054

[29] Machida S, Takizawa Y, Ishimaru M, Sugita Y, Sekine S, Nakayama J-I, Wolf M, Kurumizaka H. 2018. Structural basis of heterochromatin formation by human HP1. Mol Cell 69:385–397.e8. doi:10.1016/j.molcel.2017.12.011. 29336876

[30] Roach RJ, Garavís M, González C, Jameson GB, Filichev VV, Hale TK. 2020. Heterochromatin protein 1α interacts with parallel RNA and DNA G-quadruplexes. Nucleic Acids Res 48:682–693. doi:10.1093/nar/gkz1138. 31799602 PMC6954420

[31] Kloetgen A, Duggimpudi S, Schuschel K, Hezaveh K, Picard D, Schaal H, Remke M, Klusmann J-H, Borkhardt A, McHardy AC, Hoell JI. 2020. YBX1 indirectly targets heterochromatin-repressed inflammatory response-related apoptosis genes through regulating CBX5 mRNA. Int J Mol Sci 21:4453. doi:10.3390/ijms21124453. 32585856 PMC7352269

[32] Sun Y, Wang X, Bu X. 2021. LINC02381 contributes to cell proliferation and hinders cell apoptosis in glioma by transcriptionally enhancing CBX5. Brain Res Bull 176:121–129. doi:10.1016/j.brainresbull.2021.07.009. 34274429

[33] Aubrey BJ, Kelly GL, Janic A, Herold MJ, Strasser A. 2018. How does p53 induce apoptosis and how does this relate to p53-mediated tumour suppression? Cell Death Differ 25:104–113. doi:10.1038/cdd.2017.169. 29149101 PMC5729529

[34] Swanson KV, Deng M, Ting JP-Y. 2019. The NLRP3 inflammasome: molecular activation and regulation to therapeutics. Nat Rev Immunol 19:477–489. doi:10.1038/s41577-019-0165-0. 31036962 PMC7807242

[35] Jia X, Jia L, Mo L, Yuan S, Zheng X, He J, Chen V, Guo Q, Zheng L, Yuan Q, Xu X, Zhou X. 2019. Berberine ameliorates periodontal bone loss by regulating gut microbiota. J Dent Res 98:107–116. doi:10.1177/0022034518797275. 30199654

[36] Wang Y, Chen X, Chen X, Zhou Z, Xu W, Xu F, Zhang S. 2019. AZD8835 inhibits osteoclastogenesis and periodontitis-induced alveolar bone loss in rats. J Cell Physiol 234:10432–10444. doi:10.1002/jcp.27711. 30652303

[37] Kirst ME, Li EC, Alfant B, Chi Y-Y, Walker C, Magnusson I, Wang GP. 2015. Dysbiosis and alterations in predicted functions of the subgingival microbiome in chronic periodontitis. Appl Environ Microbiol 81:783–793. doi:10.1128/AEM.02712-14. 25398868 PMC4277562

[38] Hajishengallis G, Liang S, Payne MA, Hashim A, Jotwani R, Eskan MA, McIntosh ML, Alsam A, Kirkwood KL, Lambris JD, Darveau RP, Curtis MA. 2011. Low-abundance biofilm species orchestrates inflammatory periodontal disease through the commensal microbiota and complement. Cell Host Microbe 10:497–506. doi:10.1016/j.chom.2011.10.006. 22036469 PMC3221781

[39] Socransky SS, Haffajee AD, Cugini MA, Smith C, Kent RL. 1998. Microbial complexes in subgingival plaque. J Clin Periodontol 25:134–144. doi:10.1111/j.1600-051x.1998.tb02419.x. 9495612

[40] Mysak J, Podzimek S, Sommerova P, Lyuya-Mi Y, Bartova J, Janatova T, Prochazkova J, Duskova J. 2014. *Porphyromonas gingivalis*: major periodontopathic pathogen overview. J Immunol Res 2014:476068. doi:10.1155/2014/476068. 24741603 PMC3984870

[41] Bui FQ, Almeida-da-Silva CLC, Huynh B, Trinh A, Liu J, Woodward J, Asadi H, Ojcius DM. 2019. Association between periodontal pathogens and systemic disease. Biomed J 42:27–35. doi:10.1016/j.bj.2018.12.001. 30987702 PMC6468093

[42] Dominy SS, Lynch C, Ermini F, Benedyk M, Marczyk A, Konradi A, Nguyen M, Haditsch U, Raha D, Griffin C, Holsinger LJ, Arastu-Kapur S, Kaba S, Lee A, Ryder MI, Potempa B, Mydel P, Hellvard A, Adamowicz K, Hasturk H, Walker GD, Reynolds EC, Faull RLM, Curtis MA, Dragunow M, Potempa J. 2019. *Porphyromonas gingivalis* in Alzheimer’s disease brains: evidence for disease causation and treatment with small-molecule inhibitors. Sci Adv 5:eaau3333. doi:10.1126/sciadv.aau3333. 30746447 PMC6357742

[43] Figuero E, Han YW, Furuichi Y. 2020. Periodontal diseases and adverse pregnancy outcomes: mechanisms. Periodontol 2000 83:175–188. doi:10.1111/prd.12295. 32385886

[44] Orlandi M, Graziani F, D'Aiuto F. 2020. Periodontal therapy and cardiovascular risk. Periodontol 2000 83:107–124. doi:10.1111/prd.12299. 32385887

[45] Ho M-H, Chen C-H, Goodwin JS, Wang B-Y, Xie H. 2015. Functional advantages of *Porphyromonas gingivalis* vesicles. PLoS One 10:e0123448. doi:10.1371/journal.pone.0123448. 25897780 PMC4405273

[46] Xie H. 2015. Biogenesis and function of *Porphyromonas gingivalis* outer membrane vesicles. Future Microbiol 10:1517–1527. doi:10.2217/fmb.15.63. 26343879 PMC4603655

[47] Yang WW, Guo B, Jia WY, Jia Y. 2016. *Porphyromonas gingivalis*-derived outer membrane vesicles promote calcification of vascular smooth muscle cells through ERK1/2-RUNX2. FEBS Open Bio 6. doi:10.1002/2211-5463.12151. PMC5324769 28255538

[48] He Y, Shiotsu N, Uchida-Fukuhara Y, Guo J, Weng Y, Ikegame M, Wang Z, Ono K, Kamioka H, Torii Y, Sasaki A, Yoshida K, Okamura H. 2020. Outer membrane vesicles derived from *Porphyromonas gingivalis* induced cell death with disruption of tight junctions in human lung epithelial cells. Arch Oral Biol 118:104841. doi:10.1016/j.archoralbio.2020.104841. 32717445

[49] Seyama M, Yoshida K, Yoshida K, Fujiwara N, Ono K, Eguchi T, Kawai H, Guo J, Weng Y, Haoze Y, Uchibe K, Ikegame M, Sasaki A, Nagatsuka H, Okamoto K, Okamura H, Ozaki K. 2020. Outer membrane vesicles of *Porphyromonas gingivalis* attenuate insulin sensitivity by delivering gingipains to the liver. Biochim Biophys Acta Mol Basis Dis 1866:165731. doi:10.1016/j.bbadis.2020.165731. 32088316

[50] Turner L, Bitto NJ, Steer DL, Lo C, D’Costa K, Ramm G. 2018. Helicobacter pylori outer membrane vesicle size determines their mechanisms of host cell entry and protein content. Front Immunol 9:1466. doi:10.3389/fimmu.2018.01466. 30013553 PMC6036113

[51] Fleetwood AJ, Lee MKS, Singleton W, Achuthan A, Lee M-C, O'Brien-Simpson NM, Cook AD, Murphy AJ, Dashper SG, Reynolds EC, Hamilton JA. 2017. Metabolic remodeling, inflammasome activation, and pyroptosis in macrophages stimulated by *Porphyromonas gingivalis* and its outer membrane vesicles. Front Cell Infect Microbiol 7:351. doi:10.3389/fcimb.2017.00351. 28824884 PMC5543041

[52] Gao Y, Han M, Shang S, Wang H, Qi LS. 2021. Interrogation of the dynamic properties of higher-order heterochromatin using CRISPR/dCas9. Mol Cell 81:4287–4299.e5. doi:10.1016/j.molcel.2021.07.034. 34428454 PMC8541924

[53] Ola MS, Nawaz M, Ahsan H. 2011. Role of Bcl-2 family proteins and caspases in the regulation of apoptosis. Mol Cell Biochem 351:41–58. doi:10.1007/s11010-010-0709-x. 21210296

[54] Wu C, Zhang J. 2020. Long non-coding RNA LOXL1-AS1 sponges miR-589-5p to up-regulate CBX5 expression in renal cell carcinoma. Biosci Rep 40:BSR20200212. doi:10.1042/BSR20200212. 33185692 PMC7670581

[55] Jiang X, Wang L, Xie S, Chen Y, Song S, Lu Y, Lu D. 2020. Long noncoding RNA MEG3 blocks telomerase activity in human liver cancer stem cells epigenetically. Stem Cell Res Ther 11:518. doi:10.1186/s13287-020-02036-4. 33256840 PMC7706068

[56] Jiang W-Q, Zhong Z-H, Nguyen A, Henson JD, Toouli CD, Braithwaite AW, Reddel RR. 2009. Induction of alternative lengthening of telomeres-associated PML bodies by p53/p21 requires HP1 proteins. J Cell Biol 185:797–810. doi:10.1083/jcb.200810084. 19468068 PMC2711592

[57] Kaya-Okur HS, Wu SJ, Codomo CA, Pledger ES, Bryson TD, Henikoff JG, Ahmad K, Henikoff S. 2019. CUT&Tag for efficient epigenomic profiling of small samples and single cells. Nat Commun 10:1930. doi:10.1038/s41467-019-09982-5. 31036827 PMC6488672

[58] Lyu J, Shao R, Kwong Yung PY, Elsässer SJ. 2022. Genome-wide mapping of G-quadruplex structures with CUT&Tag. Nucleic Acids Res 50:e13. doi:10.1093/nar/gkab1073. 34792172 PMC8860588

[59] Xu T, Dong Q, Luo Y, Liu Y, Gao L, Pan Y, Zhang D. 2021. *Porphyromonas gingivalis* infection promotes mitochondrial dysfunction through Drp1-dependent mitochondrial fission in endothelial cells. Int J Oral Sci 13:28. doi:10.1038/s41368-021-00134-4. 34475379 PMC8413291

[60] Farrugia C, Stafford GP, Murdoch C. 2020. *Porphyromonas gingivalis* outer membrane vesicles increase vascular permeability. J Dent Res 99:1494–1501. doi:10.1177/0022034520943187. 32726180 PMC7684789

[61] Jönsson D, Nebel D, Bratthall G, Nilsson B-O. 2011. The human periodontal ligament cell: a fibroblast-like cell acting as an immune cell. J Periodontal Res 46:153–157. doi:10.1111/j.1600-0765.2010.01331.x. 21118418

