# Supplementary material for: Porphyromonas gingivalis Outer Membrane Vesicles Promote Apoptosis via msRNA-Regulated DNA Methylation in Periodontitis
Source: Microbiol Spectr. 2023 Jan 11;11(1):e03288-22. doi: 10.1128/spectrum.03288-22 (PMC9927323; doi:10.1128/spectrum.03288-22)
Supplement: Supplemental file 1 — Supplemental material. Download spectrum.03288-22-s0001.pdf, PDF file, 7.5 MB [file spectrum.03288-22-s0001.pdf]

Fig. S1

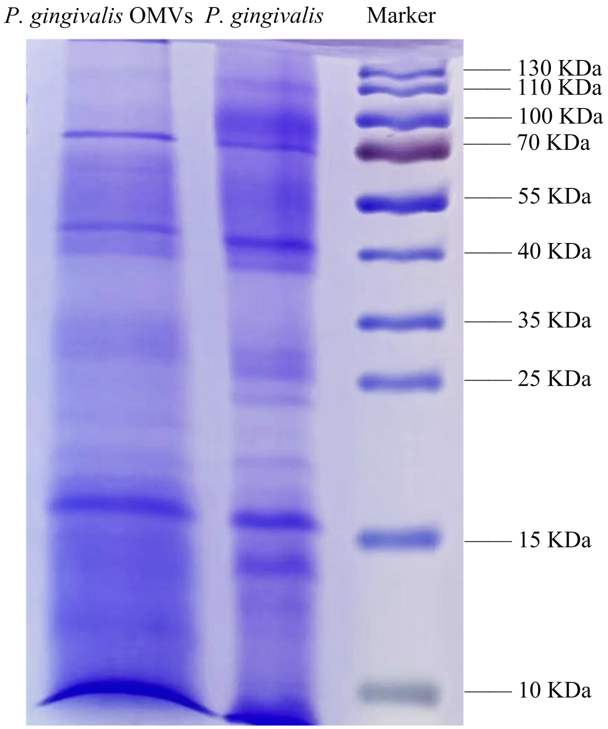

Fig. S1 Coomassie Blue Staining of *P. gingivalis* OMVs and *P. gingivalis*.

Fig. S2

A B C

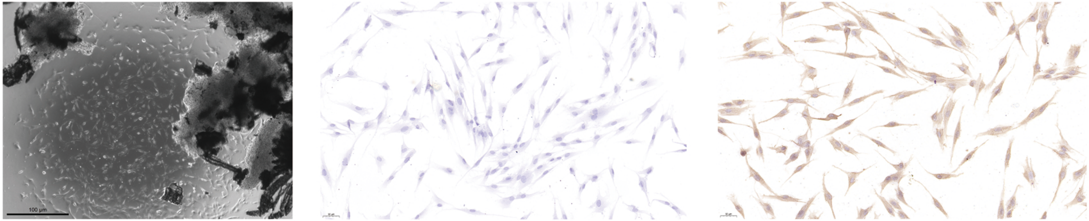

Fig. S2 Characterization of hPDLCs. (A) Primary tissue culture under light microscopy. (B) Immunohistochemical staining showed negative expression of keratin. (C) Immunohistochemical staining showed positive expression of vimentin.

Fig. S3

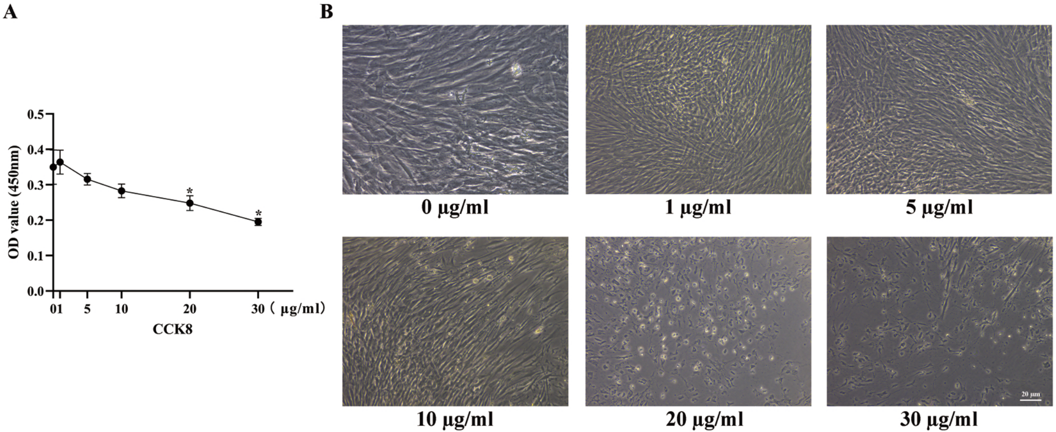

Fig. S3 The effect of different concentration of *P. gingivalis* OMVs on the viability of hPDLCs. (A) hPDLCs treated with *P. gingivalis* OMVs (0-30 μg/ml) for 24h and viability measured by CCK-8 assay. (B) Representative images taken by light microscopy.

Fig. S4

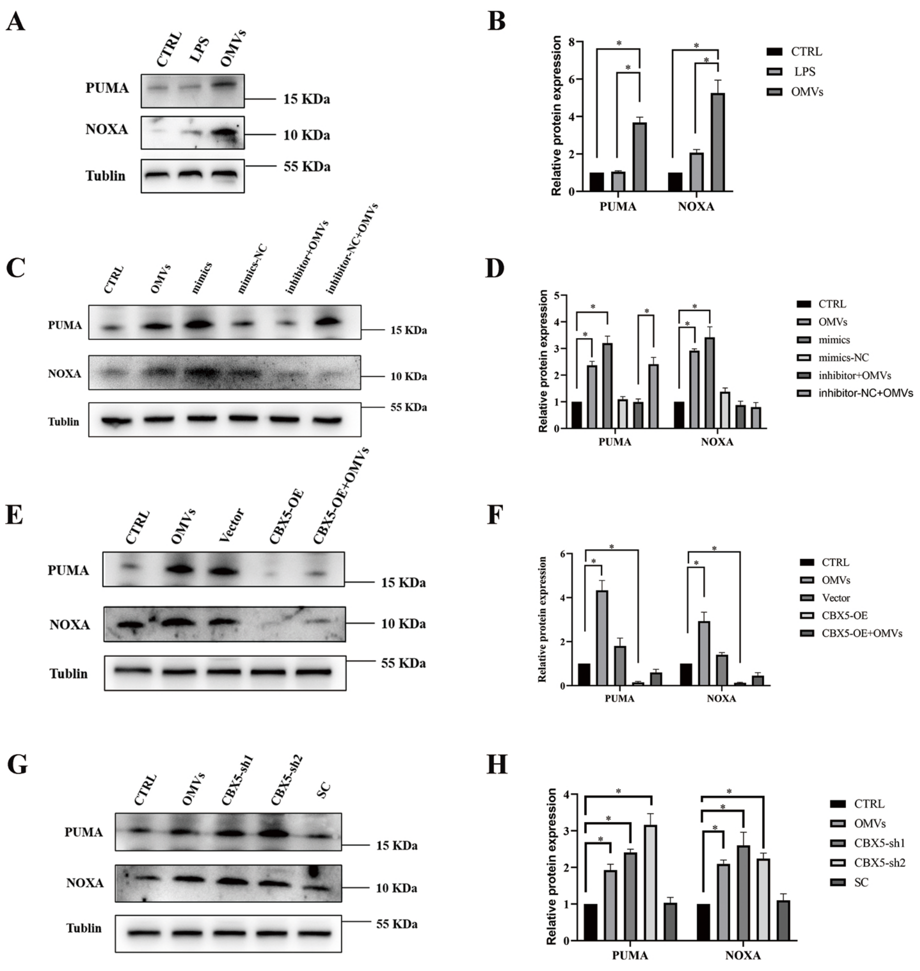

Figure. S4 Western Blot and the densitometric analyses of PUMA and NOXA expression. *$p < 0.05$.

Fig. S5

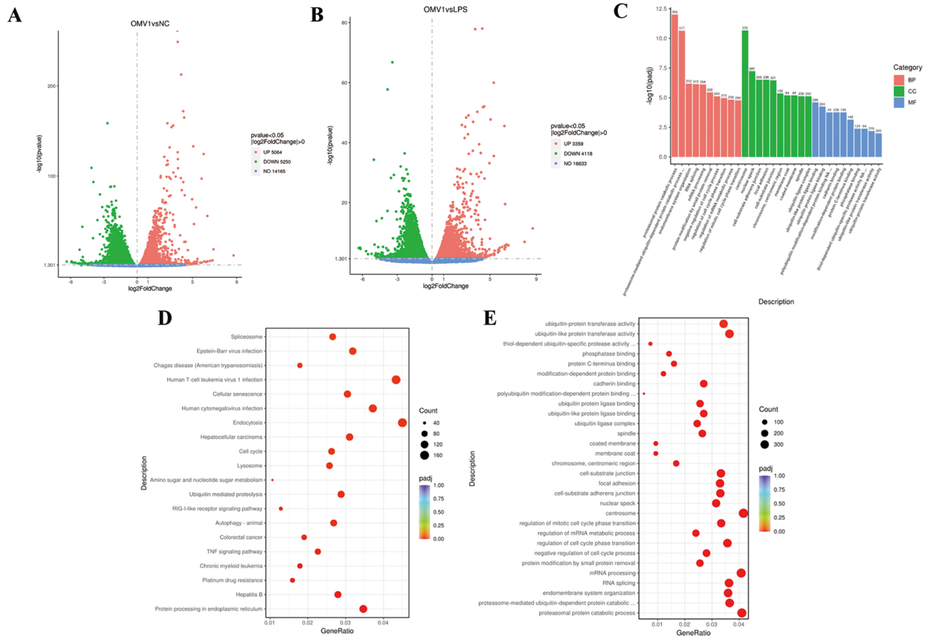

Fig. S5 Transcriptome sequencing analysis of hPDLCs administrated *P. gingivalis* OMVs. (A-B) Volcano plot of *P. gingivalis* OMVs agains control and *P. gingivalis* LPS. (C) GO enrichment analysis of *P. gingivalis* OMVs. (D-E) KEGG pathway analysis of *P. gingivalis* OMVs.
